# Bio‐Inspired Oligocellulose‐Regulated Ice‐Water Interfaces Enable Sustained Ion Transport in Frozen Aqueous Zinc Batteries

**DOI:** 10.1002/advs.77291

**Published:** 2026-08-24

**Authors:** Zeyu Zhu, Jingxuan Pan, Haoran Ma, Boya Yuan, Cristina Carucci, Sunghak Park, Chong Gao, Minmin Liang, Kaiqi Li, Wei Li, Dan Wang, Xiaoting Chen, Zhiyuan He

**Affiliations:** ^1^ School of Materials Science and Engineering Beijing Institute of Technology Beijing P. R. China; ^2^ Department of Chemical and Geological Sciences University of Cagliari & Center For Colloid and Surface Science (CSGI) Monserrato Cagliari Italy; ^3^ Department of Future Energy Engineering Sungkyunkwan University Suwon Republic of Korea; ^4^ Tangshan Research Institute Beijing Institute of Technology Beijing P. R. China

**Keywords:** antifreeze glycoproteins, aqueous zinc ion batteries, eco‐friendly saccharide additive, ice recrystallization inhibition, oligocellulose

## Abstract

While highly concentrated salts and organic components enhance the subzero performance of zinc batteries by improving electrolyte fluidity and conductivity, their high cost and environmental pollution negate the inherent sustainability and economy of aqueous electrolytes. Herein, inspired by the ice‐inhibition mechanism of natural antifreeze glycoproteins, we designed oligomeric cellulose with an average degree of polymerization of 8 (OC‐8). At trace concentration, OC‐8 effectively suppresses ice recrystallization and induces a fourfold increase in inter‐ice water channels. In situ confocal fluorescence microscopy confirms that this interconnected network boosts ion transport by nearly an order of magnitude in cryogenic electrolytes. Complementary molecular dynamics simulations coupled with CHILL^+^ structural analysis reveal that OC‐8 adsorbs at the ice‐water interface, perturbs interfacial hydrogen‐bond ordering, and stabilizes extended quasi‐liquid domains, enabling efficient Zn^2+^ transport at low temperatures. Leveraging this antifreeze mechanism, Zn||Zn cells achieve stable cycling for over 1200 h at −30°C. The full Zn||NH_4_^+^‐V_2_O_5_ cell sustains remarkable long‐term stability over 3000 cycles, simultaneously suppressing zinc dendrite growth and detrimental side reactions. This work provides a fundamental mechanistic insight into saccharide‐based antifreeze agents and presents a bio‐inspired strategy for engineering an eco‐friendly, high‐performance, and durable antifreeze aqueous electrolyte.

## Graphical abstract

Inspired by antifreeze glycoproteins (AFGPs), we synthesized a type of oligomeric cellulose (OC‐8) to inhibit ice formation and enlarge the inter‐ice water channel for transportation. Typical confocal fluorescence experiments display the expansion of the ice‐water interface for zinc‐ion transport. This work verifies the correlation between frozen electrolyte regulation and ionic conductivity enhancement under low temperatures.

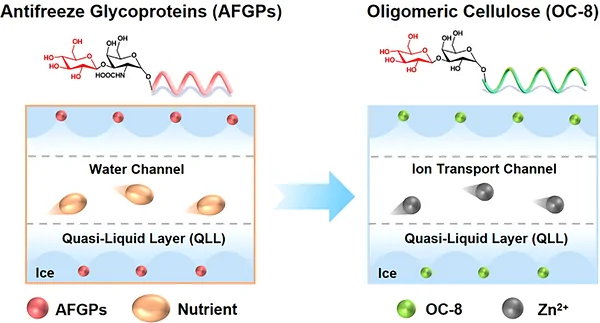

## 1 Introduction

Aqueous electrolytes, usually comprising typically more than 70 wt.% water, present considerable advantages in safety and sustainability owing to their inherent characteristics (elevated dielectric constant, affordability, and environmental benignity), and are central to next‐generation electrochemical energy storage, particularly in the realm of aqueous rechargeable batteries [1, 2, 3]. Nonetheless, these benefits are not guaranteed when employed at subzero conditions, as they remain susceptible to faulty operation and experience catastrophic incidents primarily caused by freezing [4, 5]. To mitigate issues above, exploration of electrolyte additives has been perceived as an indispensable strategy for enhancing low‐temperature performance for aqueous rechargeable batteries, primarily due to their “minimal dosage” and “rapid effect” [6, 7, 8, 9, 10]. Efficient additives for low‐temperature aqueous electrolytes typically need to satisfy the requirements of low melting point, miscibility with water and electrodes, and robust electrochemical and thermodynamic stability [9]. In line with these principles, previous endeavors have explored various additives such as dimethyl sulfoxide (DMSO) [5, 11], acetonitrile [12], and organic compounds like betaine [13], tetramethylurea [14]. In addition, thorough research has also been conducted on polymers like polyethylene glycol (PEG) [15, 16], and polyvinyl alcohol (PVA) [17]. These additives have proven to competitively form hydrogen bonds with water molecules and markedly lower the freezing point of water [18]; however, concerns persist regarding high cost and toxicity [19, 20]. Hence, the development of applicable low‐cost and eco‐friendly low‐temperature aqueous electrolyte additives, along with elucidating the mechanism through which aqueous electrolytes may potentially be protected from freezing, is of paramount importance.

Naturally occurring antifreeze glycoproteins (AFGPs) in cold‐acclimatized fish and microorganisms exemplify a striking case of convergent evolution that enables survival under extremely low‐temperature conditions [21, 22]. Thorough research has revealed that the essential functional element of AFGPs is the glycan moiety, composed of saccharide units (as illustrated in Figure 1a) [23]. These functional saccharide units facilitate interactions with water molecules and/or binding to ice crystal surfaces, thereby inhibiting ice crystal growth and aggregation, and expanding water channels for nutrient transport at subzero temperatures (Figure 1a) [24, 25]. Likewise, frost‐resistant plants, such as cedar, have independently evolved to produce active soluble saccharides like sucrose, glucose, and specialized cellulose to modify the physicochemical properties of intracellular fluids, preserving cell stability in severely cold conditions [26, 27]. Saccharide‐based additives, inspired by these biological processes, have been widely investigated for cryopreservation of cells and organs [28, 29]. Researchers also reported enhanced low‐temperature resistance and cyclic performance of zinc‐ion batteries with saccharide‐modified aqueous electrolytes [30, 31, 32, 33, 34]. For instance, Chen et al. reported a sucrose biomolecule aqueous electrolyte for aqueous zinc ion batteries boosted affording high‐performance at −20°C due to the steric‐hindrance effect [35]. Latest studies have shown that aqueous electrolytes with trace α‐D‐glucose implement stable cycling at −25°C via dual remodeling effect, altering both the Zn^2+^ primary solvent shell and the Zn anode surface [36]. Despite promising results, the fundamental mechanism of saccharide architectures and their influence on ion transport in icy electrolytes remains insufficiently understood. A deeper mechanistic clarity is essential for transitioning from empirical screening to a predictive and systematic approach to electrolytes design [37, 38].

**FIGURE 1:**
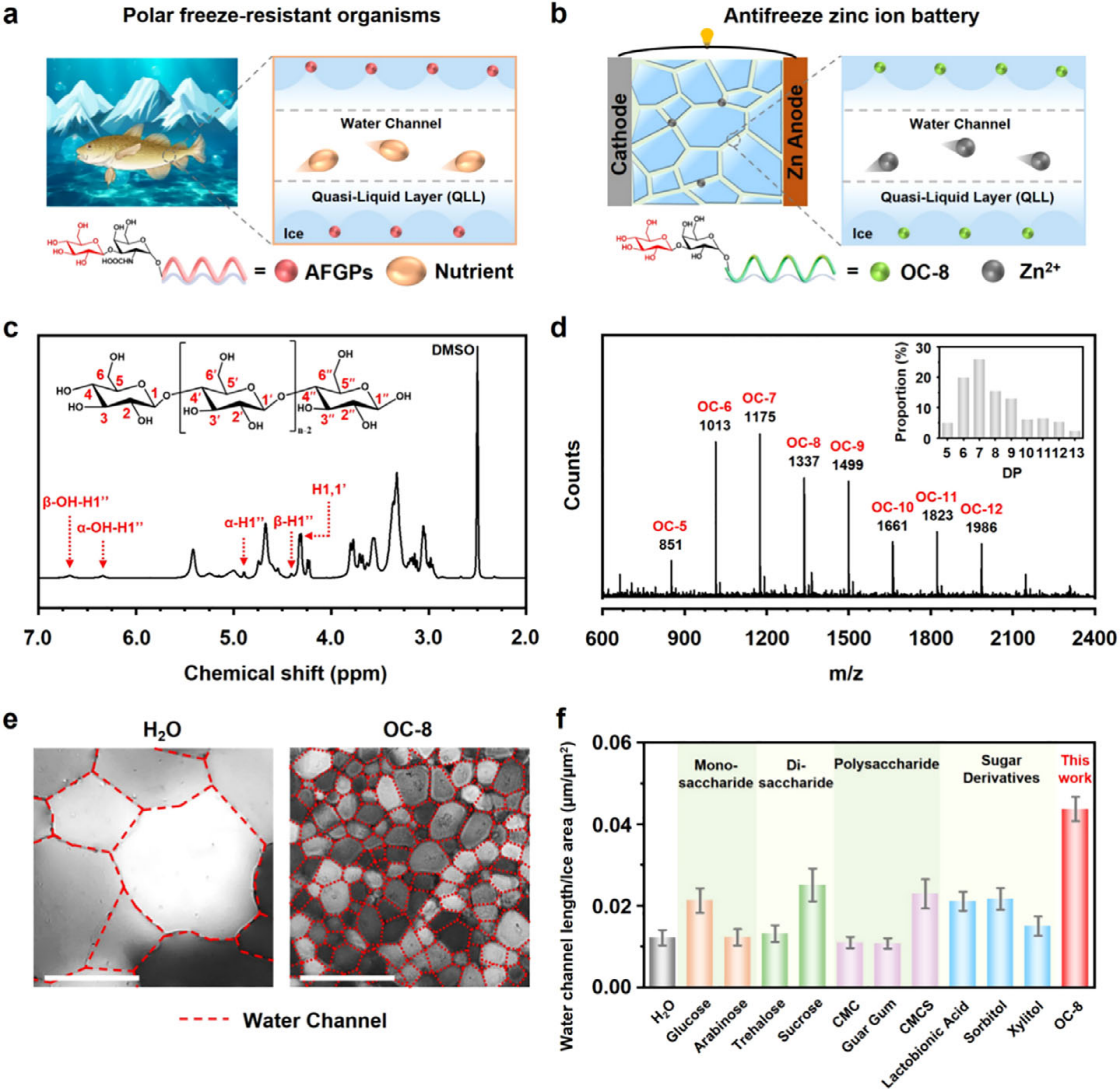
(a) Structure of antifreeze glycoproteins (AFGPs) from nototheniid fish and their ice‐inhibition mechanism. (b) Schematic diagram of the biomimetic strategy in this work. Oligocellulose (OC‐8) with ice formation inhibiting capability could provide more water channels to enhance zinc ion transport and boost the efficiency of AZIBs. (c) ^1^H NMR spectrum of OC‐8 in DMSO. Characteristic chemical shifts related to the specific bonds are annotated on the chemical structure in the inset. (d) Positive‐ion mode MALDI counts as a function of mass‐to‐charge (m/z) ratio for the oligocellulose. The molecular mass quantity and corresponding degree of polymerization are noted on each mainline. The inset illustrates the proportion of OC‐8 in the mixture of cellulose. (e) Optical micrographs of ice crystal and inter‐ice water channel of water and OC‐8 solution (10 mg mL^−1^) after annealing for 40 min at −8°C. Scale bar: 100 µm. (f) Quantitative calculation of the length of water channels between ice. The error bar indicates that at least five tests have been conducted.

Herein, inspired by the bioprocess of ice growth inhibition achieved by AFGPs, we successfully designed and synthesized oligomeric cellulose, characterized by a narrow degree of polymerization distribution peaked at 8 (OC‐8). This precise molecular architecture functions as a highly potent ice recrystallization inhibition (IRI) agent, providing more water channels for ion transport in AZIBs (Figure 1b). Comparative analysis across a broad spectrum of common saccharides (including mono‐, di‐, poly‐saccharides, and sugar derivatives) unequivocally demonstrates that OC‐8 is superior in suppressing ice formation and recrystallization, even at extremely low concentrations. IRI and nanoliter osmometry experiments initially confirmed that the synthetic OC‐8 effectively regulates the structure of frozen electrolyte. Further, confocal microscopy and electrochemical impedance spectroscopy (EIS) provided crucial visual and electrochemical evidence, revealing that OC‐8 significantly expands the quasi‐liquid layers (QLLs) within the frozen matrix, thereby facilitating robust ion transport at subzero temperatures. Molecular dynamics (MD) simulations were conducted in this research. The CHILL+ algorithm was employed to quantitatively analyze the evolution of ice crystal growth at the molecular level. We discovered that OC‐8 could adsorb on the surface of ice crystals and obstruct ice growth. This occurrence is known as the adsorption‐inhibition phenomenon. Crucially, a trace amount (10 mg mL^−1^) of OC‐8 modified zinc trifluoromethanesulfonate (ZOTf) electrolyte promotes stable operation of Zn||Zn symmetric batteries to operate stably for over 1200 h at −30°C. Notably, the full cell experiments unveil that the Zn||NH_4_^+^‐V_2_O_5_ battery maintains an exceptional steady cycle life of over 3000 cycles under −30°C.

## 2 Results and Discussion

Inspired by the ice‐binding mechanism of antifreeze glycoproteins (AFGPs), we utilized a type of oligocellulose with a relatively narrow molecular weight distribution. Of note, precise control over the degree of polymerization is crucial, as the degree of polymerization profoundly affects both the water solubility and the binding affinity to ice surfaces (Figure 1a). Fourier transform infrared spectroscopy (FT‐IR) and x‐ray diffraction (XRD) confirmed that the synthesized products retained the typical cellulose backbone (Figures S1 and S2), and the structural profile is consistent with typical oligocellulose structures [39]. Further analysis using proton nuclear magnetic resonance spectroscopy (^1^H‐NMR) (Figure 1c) and matrix‐assisted laser desorption/ionization (MALDI) mass spectrometry (Figure 1d) verified that the dominant component was oligocellulose with an average degree of polymerization centered at 8 (denoted as OC‐8 subsequently).

We first evaluated the anti‐freezing capacity of OC‐8 using the ice recrystallization inhibition (IRI) assay via the splat cooling method (refer to the Supporting Information for the experimental details). The aqueous solution, with and without OC‐8, was annealed for 40 min at −8°C. The microscopic image in S3 clearly showed that the ice crystals in pure water coarsened rapidly to reach an average grain size of 126 µm. In stark contrast, the inclusion of 10 mg mL^−1^ of OC‐8 dramatically reduced the grain size to approximately 33 µm, demonstrating potent IRI capability. Therefore, more water channels can be formed between small ice crystals, as shown in Figure 1e. Additionally, a comparative study was conducted across ten different types of previously reported saccharides, including monosaccharide (glucose), disaccharide (sucrose), polysaccharide (carboxymethyl cellulose, CMC), and sugar derivatives. Through IRI experiments, we calculate the length of water channels between ice. Figure S4 and 1f unequivocally demonstrated that OC‐8 is superior in suppressing ice recrystallization, which efficaciously expands the length of water channels between ice (at the same concentration of 10 mg mL^−1^). Compared to the blank aqueous solution, the water channel length between ice in the solution with OC‐8 added at low temperatures is four times longer and much longer than other selected sugar additives, highlighting its exceptional IRI effectiveness. We also employed a YASN nanoliter osmometer to investigate the morphology modification ability of the synthetic OC‐8 on a single ice crystal (refer to the Supporting Information for the experimental details). The results indicated that the synthetic OC‐8 slightly altered the crystal habit from circular to hexagonal at a moderate supercooling of 0.10°C (Figures S5 and S6). This transition suggests a preferential adsorption of OC‐8 on specific ice crystal faces, analogous to the ice‐binding capability reported from antifreeze glycoproteins (AFGPs). Collectively, these findings demonstrate that the synthetic OC‐8 effectively modulates ice growth kinetics even at trace concentrations.

### 2.1 Ice‐Inhibition Capability and Ion‐Transport Efficiency

To elucidate the relationship between the observed IRI capability translates of OC‐8 and its practical electrochemical performance, we investigated the effect of OC‐8 on zinc ion (Zn^2+^) transport within a frozen electrolyte. As shown in Figure 2a, zinc salt solution containing OC‐8 was frozen on a precooled substrate (−80°C), followed by the deposition of a second ice layer incorporating the Zn^2+^ indicator Zinpyr‐1. Upon excitation with blue light (λ = 488 nm), the fluorescent indicator (Zinpyr‐1) undergoes a significant enhancement in quantum yield upon binding to Zn^2+^, resulting in green fluorescence. This optical signal serves as a precise reporter for the spatiotemporal distribution of Zn^2+^, allowing for the direct tracking of zinc ion migration within the cryogenic electrolyte (as illustrated in Figure 2a) [40]. Confocal fluorescence microscopy revealed that the presence of OC‐8 promotes a dense network of fluorescent pathways, confirming the existence of unfrozen aqueous channels surrounding the ice crystals, which enable efficient Zn^2+^ migration (Figure 2b). Based on the number of fluorescent channels, we roughly conclude that Zn^2+^ ion transport rate in ice with 10 mg mL^−1^ OC‐8 was about ten times that of the blank one. (Figure 2c).

**FIGURE 2:**
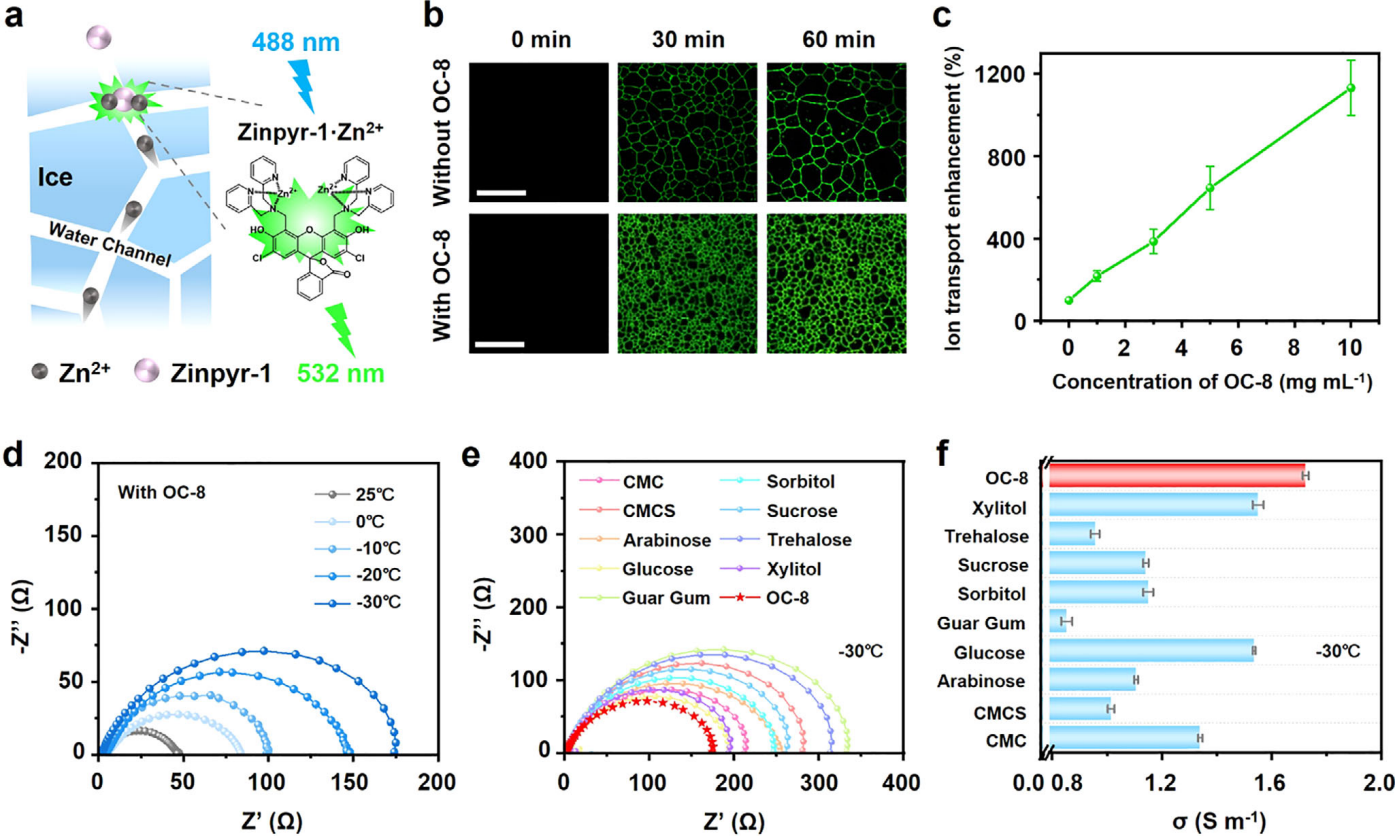
Experimental design of ion transport channel variation and ion conductivity comparison. (a) Schematic diagram of zinc‐ion transport experiment in ice‐water interface channel. (b) Typical confocal fluorescence images of the zinc ion transport process in the frozen state with and without OC‐8. Scale bar: 100 µm. (c) Calculation of Zn^2+^ transport enhancement rate under different concentrations of OC‐8. (d) Impedance curve of zinc ion electrolyte at gradient temperature ranging from 25°C to −30°C after adding 10 mg mL^−1^ OC‐8. Comparison of (e) impedance curves and (f) ionic conductivity of zinc ion electrolytes after adding different types of saccharides (concentration: 10 mg mL^−1^) at −30°C.

This visual evidence was directly correlated with electrochemical performance. 1 m solution of zinc trifluoromethanesulfonate (ZOTf) electrolytes containing 10 mg mL^−1^ of OC‐8 and aforementioned saccharides, respectively, were prepared. The macroscopic images and differential scanning calorimetry (DSC) tests at different temperatures indicated that the electrolyte was frozen and lacked fluidity at such a low temperature of −30°C (Figures S7 and S8). Electrochemical Impedance Spectroscopy (EIS) confirmed that, upon cooling from 25°C to −30°C, the ionic resistance of OC‐8 modified electrolyte increased gradually and stabilized at 174 Ω (Figure 2d). The ionic resistance significantly outperformed that of electrolytes containing all other comparative saccharides. The result here further verifies the efficiency of OC‐8 in preserving conductive channels for frozen electrolytes with extremely low concentrations (Figure 2e, f). The physical properties of OC‐8 were further evaluated using a 10 mg mL^−1^ aqueous solution. At 25°C, the solution exhibits an ionic conductivity of 6.18 mS cm^−1^ and pronounced shear‐thinning behavior, with its apparent viscosity decreasing markedly as the shear rate increases (Figure S9).

### 2.2 Theoretical Calculation Level of OC‐8 Increasing the Ion Transport Channels

To elucidate the molecular mechanism underlying the ice inhibition of OC‐8, molecular dynamics (MD) simulations were conducted at 243 K to probe the structural evolution at the ice‐water interface. The simulations reveal that the OC‐8 molecule preferentially adsorbs onto the interface and disrupts the ordered growth of ice, as shown in Figure 3a (see Figure S10 for additional details). As the simulation proceeds from 0 to 100 ns, the ice front becomes increasingly curved, which is a manifestation of the Gibbs‐Thomson effect [4], where enhanced interfacial curvature depresses the local freezing point and suppresses crystal propagation. This curved interface is accompanied by the stabilization of a quasi‐liquid layer (QLL), which is a partially ordered transitional region between the crystalline ice front and the bulk liquid. In the presence of OC‐8, the QLL is kinetically stabilized through the disruption of interfacial hydrogen bonding, which prevents further ice propagation and simultaneously provides a continuous medium for ion diffusion at subfreezing temperatures. Similar curvature‐driven suppression of ice growth has been reported in other antifreeze systems [41, 42, 43]. In contrast, the pure ice‐water system without OC‐8 (Figure S11) maintains a flat, stable interface throughout the simulation, highlighting the distinct interfacial disruption observed only in the presence of OC‐8. This disruption destabilizes the quasi‐liquid layer, hinders further crystallization, and offers a molecular‐level explanation for the freezing‐point depression observed experimentally.

**FIGURE 3:**
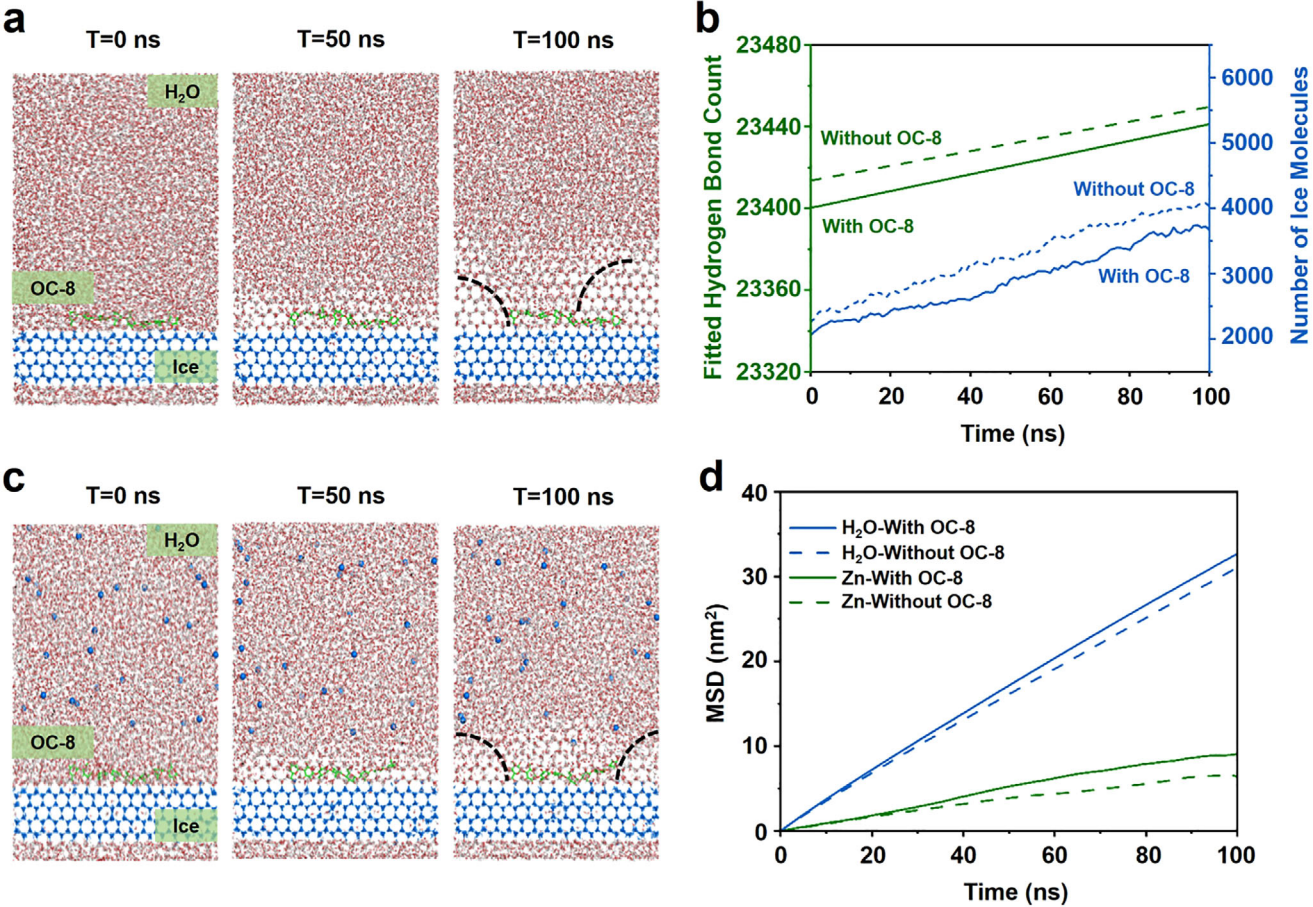
Molecular‐scale insights into ice inhibition and electrolyte behavior at −30°C. (a) Molecular dynamics (MD) snapshots of the ice slab surface in the presence of OC‐8 at 0, 50, and 100 ns. The dashed black lines indicate surface curvature influenced by the Gibbs‐Thomson effect. (b) Time evolution of the number of hydrogen bonds (left) and the number of hexagonal ice water molecules (right). (c) MD snapshots of the OC‐8 system with 30 ZnCl_2_ molecules added to model electrolyte conditions at −30°C. (d) Mean square displacement (MSD) profiles of Zn^2+^ ions and water molecules at −30°C.

The inhibitory role of OC‐8 was further evaluated by monitoring the temporal evolution of the hydrogen bond during the simulation, as shown on the left axis of Figure 3b. As crystallization progressed, the number of hydrogen bonds increased in both systems, reflecting the stronger hydrogen‐bonding network in ice compared with liquid water [42]. However, the system containing OC‐8 consistently exhibited fewer hydrogen bonds than the system without OC‐8, indicating that OC‐8 disrupts interfacial hydrogen bonding and suppresses ice growth. While hydrogen‐bond analysis provides supportive evidence of this effect, a more direct quantification was obtained using the CHILL+ algorithm [43, 44], which distinguishes ice‐like and liquid‐like water molecules along the simulation trajectory. In the presence of OC‐8, the number of hexagonal ice molecules remained consistently lower than in the system without OC‐8 (Figure 3b, right axis), confirming the suppression of ice crystallization at the molecular scale.

Efficient ion transport is essential for the performance of aqueous zinc‐ion batteries, yet electrolyte freezing at low temperatures severely impedes ionic mobility. To probe how OC‐8 mitigates this limitation, Zn^2+^ ions were introduced into the simulation system to analyze their interfacial behavior (Figure S12). Importantly, this substitution does not qualitatively affect the mechanistic conclusions, as the simulations focus on the interfacial inhibition behavior rather than exact electrolyte replication. As shown in Figure 3c (see Figure S12 for additional details), the coexistence of OC‐8 and Zn^2+^ ions maintains the interfacial curvature observed in the ion‐free system (Figure 3a), and CHILL+ analysis verifies that the ice‐inhibiting effect of OC‐8 persists in the presence of ions (Figure S13). In contrast, Zn^2+^ alone only slightly delays crystallization without altering the overall planar morphology of the interface (Figure S14).

Mean‐squared displacement (MSD) analysis (Figure 3d) reveals that both water molecules and Zn^2+^ ions exhibit substantially higher mobility in the OC‐8 system. These enhanced molecular dynamics indicate that OC‐8 not only prevents interfacial solidification but also stabilizes a dynamic quasi‐liquid layer that serves as a continuous pathway for ion transport under subfreezing conditions. These results show that OC‐8 interacts synergistically with solvated ions to create a more dynamic and conductive medium under freezing conditions, which is essential for the stable operation of aqueous zinc‐ion batteries at low temperatures. To further examine the chemical environment of the electrolyte components, XPS measurements were performed on freeze‐dried ZOTf and ZOTf‐OC‐8 electrolytes. The changes in the C 1s and O 1s components, together with the nearly unchanged Zn 2p peak positions, suggest that OC‐8 regulates the local oxygen‐containing and hydrogen‐bonding environment without substantially changing the chemical state of Zn species (Figure S15).

### 2.3 Electrochemical Characteristics of OC‐8‐modified Electrolytes Under Ambient Conditions

A series of Zn||Zn symmetric batteries, Zn||Cu asymmetric batteries and Zn||NH_4_^+^‐V_2_O_5_ (NVO) full batteries have been assembled to verify the compatibility and modification effect of OC‐8 on zinc anodes. Figure 4a illustrates the improved Coulombic efficiency (CE) of a Zn||Cu asymmetric battery with the OC‐8 modified electrolyte, which maintains a stable CE of 98.75% for over 400 cycles. In sharp contrast, asymmetric batteries prepared with 1 m ZOTf electrolyte failed rapidly after less than 70 cycles of operation (Figure S16). The enhanced cycling stability of the OC‐8 modified electrolyte is further demonstrated by the long‐term cycling performance of Zn||Zn symmetric batteries at different current densities (Figure 4b, c). Symmetric battery with electrolyte in the absence of OC‐8 failed after operating 250 h, while the battery relying on OC‐8 modified electrolyte operated stably for over 1000 h at 1 mA cm^−2^ (Figure 4b). Even when the current density was increased to 3 mA cm^−2^, as shown in Figure 4c, the battery using ZOTf‐OC‐8 electrolyte could still operate steadily for over 400 h, while the ZOTf electrolyte experienced a short circuit in less than 100 h. Consistent with these improvements, the Zn^2+^ transference number increased from 0.07 for the ZOTf electrolyte to 0.24 for the ZOTf‐OC‐8 electrolyte (Figure S17), indicating that OC‐8 increases the relative contribution of Zn^2^^+^ to ionic transport and alleviates interfacial concentration polarization.

**FIGURE 4:**
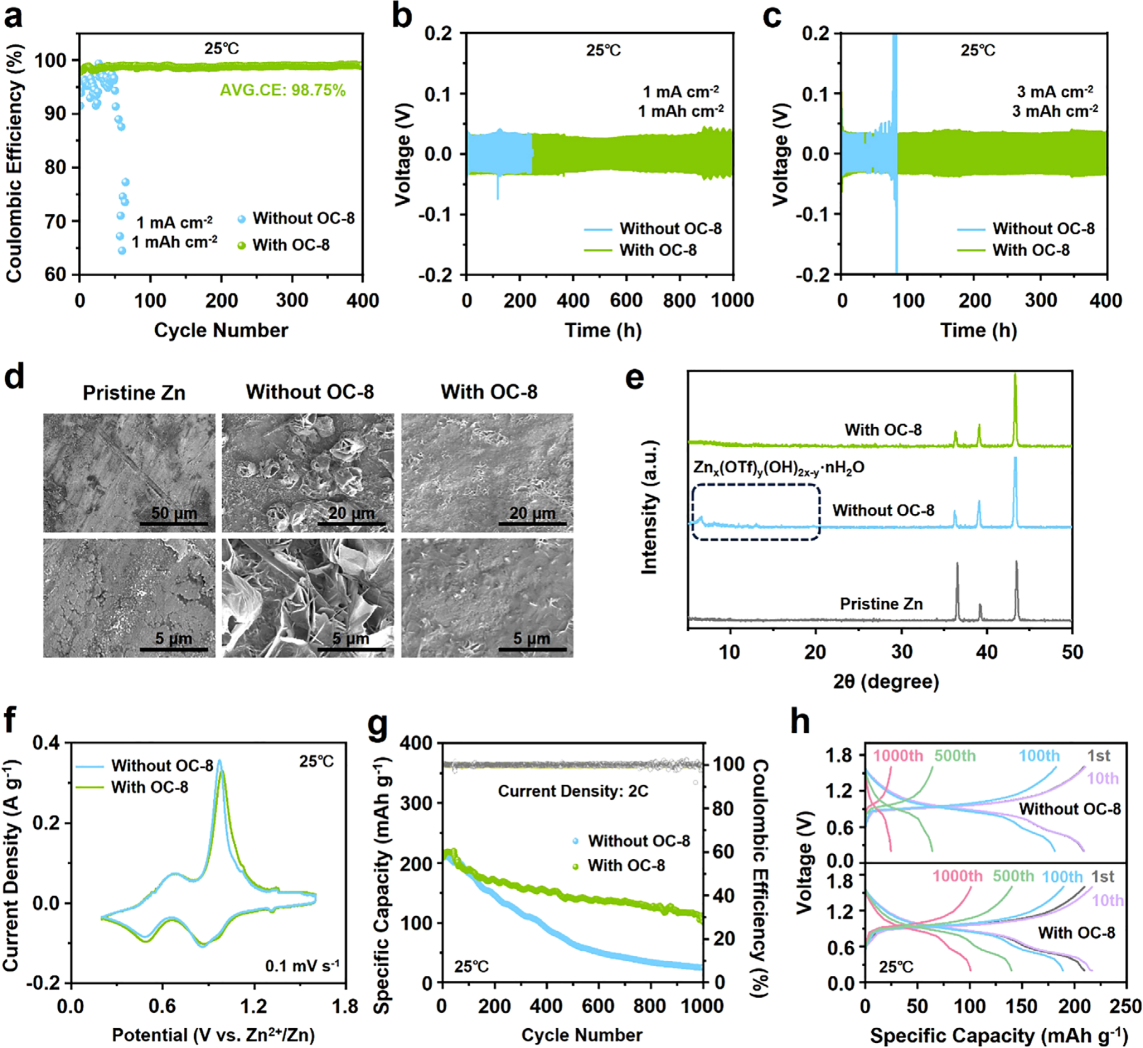
Rate and cycling performance of the battery at room temperature. (a) Coulombic efficiency of the Zn||Cu asymmetric batteries using electrolyte with and without OC‐8 at 1 mA cm^−2^/1 mAh cm^−2^. Cycling performance of Zn||Zn symmetric batteries using ZOTf and ZOTf‐OC‐8 electrolyte at (b) 1 mA cm^−2^/1 mAh cm^−2^ and (c) 3 mA cm^−2^/3 mAh cm^−2^. (d) SEM images and (e) XRD spectrum of the pristine Zn and the zinc anode of symmetrical batteries assembled with different electrolytes after running at room temperature for 20 h. (f) CV curves at the scan rate of 0.1 mV s^−1^ using electrolyte with and without OC‐8. (g) Cycling performance of the Zn||NVO full battery using both electrolytes at room temperature under a current density of 2C. (h) Related discharge–charge voltage profiles of Zn|ZOTf|NVO and Zn|ZOTf‐OC‐8|NVO batteries under different cycles.

Scanning Electron Microscopy (SEM) images in Figure 4d, e provided insights into the underlying mechanism, the OC‐8 electrolyte prevents the formation of detrimental zinc dendrites and maintains a flat anode surface after cell operation of over 20 h. This is in strong contrast to the dendrite‐covered anode observed in the cell with ZOTf. XRD analysis in Figure 4e confirmed that OC‐8 effectively suppresses undesirable side reactions. The characteristic peaks demonstrated the formation of byproducts like Zn_x_(OTf)_y_(OH)_2x‐y_·nH_2_O on zinc anode surfaces for a cell using ZOTf [14]. Furthermore, the cyclic voltammetry curves for both electrolytes with and without OC‐8, cycled from 0.2 to 1.8 V with a scan rate of 0.1 mV s^−1^, exhibited highly consistent oxidation‐reduction peaks, indicating that the incorporation of OC‐8 does not interfere with the intrinsic, reversible redox process of the anode electrode surface. The long‐term cycling performance was evaluated for a full cell utilizing electrolyte with and without OC‐8. Figure 4g, h showcase that the full cell suffered a rapid capacity decay, plummeting from an initial 220 mAh g^−1^ to approximately 28 mAh g^−1^ after 1000 cycles. In stark contrast, the cell with OC‐8 modified electrolyte demonstrated significantly enhanced stability, with its capacity decreasing to a much higher value of 112 mAh g^−1^. Our results highlight that OC‐8 not only stabilizes the Zn anode but also substantially prolongs the lifespan and capacity retention of the full battery.

### 2.4 Low‐temperature Electrochemical Performance of OC‐8 Modified Electrolyte

It is critical to estimate the correlation between the ice recrystallization inhibition (IRI) and enhanced ionic transport of the OC‐8 modified electrolyte and the resultant electrochemical performance under extreme cold conditions. Additional concentration‐dependent electrochemical measurements further showed that 10 mg mL^−1^ affords the best overall battery performance among the tested dosages and was therefore selected for the main electrochemical studies (Figure S18). Figure 5a showed the Zn||Zn symmetric cell assembled with ZOTf‐OC‐8 electrolyte maintained stable operation across a wide temperature window, from 25°C down to −30°C at 1 mA cm^−2^, whereas batteries assembled with blank electrolytes experienced rapid failure at −30°C. Figure 5b demonstrates that the Zn||Cu asymmetric battery with OC‐8 modified electrolyte could maintain an average CE of 99.85% over 500 cycles at −30°C. Further post‐mortem characterization after 50 cycles at −30°C shows that the Cu electrode cycled with the ZOTf‐OC‐8 electrolyte exhibits a more uniform surface morphology (Figure S19). XPS analysis of the corresponding Zn electrodes reveals changes in the C─O‐related contribution and a reduced relative contribution from low‐binding‐energy sulfur‐containing species after the addition of OC‐8 (Figure S20). These results support the role of OC‐8 in regulating Zn deposition and stabilizing the Zn/electrolyte interphase. The OC‐8 modified electrolyte enabled symmetric cells to sustain stable cycling for over 1200 h, directly confirming enhanced frost resistance achieved through the provision of efficient zinc ion transport channels. Remarkably, the SEM image of the surface of the Zn anode surface from the symmetrical battery under modification of OC‐8 revealed that the surface remained flat after running for 50 cycles at a low temperature of −30°C, indicating the protective effect of the oligocellulose on the Zn anode in an extreme environment (Figure S21). At −30°C, full Zn||NVO cells demonstrated high‐rate capability with specific capacity of 135.2, 122.4, 101.5, 80.2 mAh g^−1^ at a current density of 1C, 2C, 4C and 8C, corresponding to approximately 0.26, 0.52, 1.04, and 2.08 A g^−1^, respectively (Figure 5d). To clarify the structure‐property relationship, oligocellulose samples with different average degrees of polymerization, including OC‐6, OC‐8, OC‐15, and OC‐20, were comparatively evaluated at the same additive concentration of 10 mg mL^−1^. The room‐temperature Zn||Zn cycling results and the low‐temperature Zn||Zn/Zn||Cu comparison further demonstrate that OC‐8 delivers the best overall performance (Figures S22 and S23). Notably, prolonged cycling tests confirmed stable operation of the full battery with a capacity of 122 mAh g^−1^ for more than 3000 cycles at −30°C (Figure 5e). Comparative studies against over ten different saccharide additives (Figure 5f) shows that OC‐8 provides superior antifreeze and electrochemical performance at a considerably minimal loading (10 mg mL^−1^). The results underline that OC‐8 enables persistent zinc ion transport and electrode structural stability within frozen electrolytes.

**FIGURE 5:**
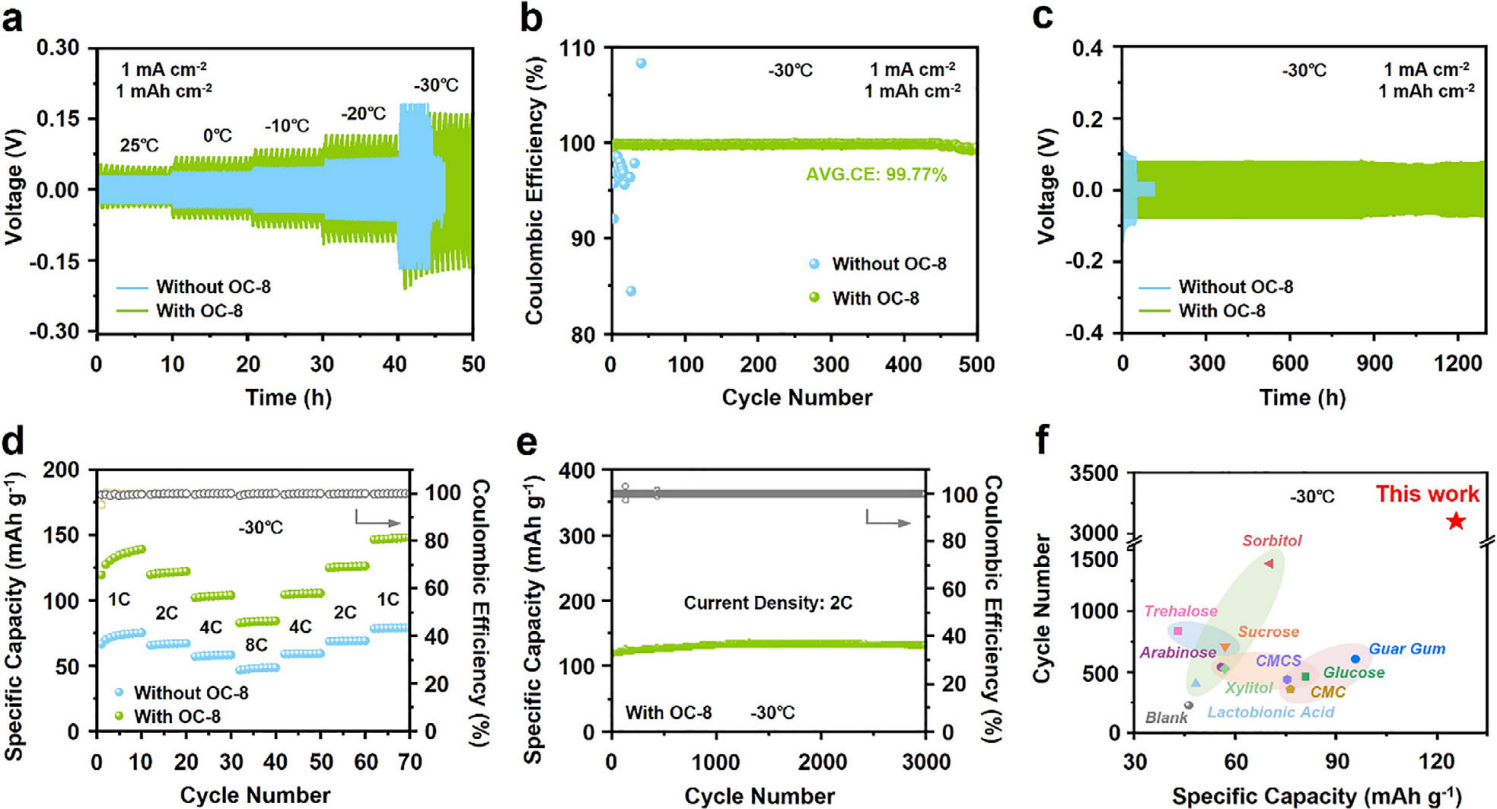
Low temperature performance of the battery assembled with OC‐8 modified electrolyte. (a) Cyclic performance of Zn||Zn symmetric batteries under gradient temperatures ranging from −30°C to 25°C at 1 mA cm^−2^/1 mAh cm^−2^. (b) Coulombic efficiency of the Zn||Cu asymmetric batteries using ZOTf and ZOTf‐OC‐8 electrolytes under −30°C. (c) Long‐term cycling performance of Zn||Zn symmetric batteries using ZOTf electrolyte with and without OC‐8 at −30°C. (d) Discharge capacities of the full batteries using different electrolytes operated under −30°C at different current densities. (e) Cycling performance of Zn||NVO full battery using OC‐8 modified electrolyte at −30°C. (f) Comparison of specific capacity and cycling performance of full batteries using electrolyte modified by different saccharides and OC‐8 (concentration: 10 mg mL^−1^) at −30°C.

## 3 Conclusion

To conclude, inspired by the antifreeze glycoproteins (AFGPs) in polar freeze‐resistant organisms, we synthesized oligomeric cellulose (OC‐8) with the ability to inhibit ice recrystallization to enlarge the inter‐ice water channel for transport under low temperatures. Related confocal fluorescence experiments have shown that solutions containing trace amounts of such AFGPs mimics can significantly expand the ice‐water interface layer for zinc ion transport and improve the ionic conductivity at low temperatures. Molecular dynamics simulations elucidate, at the atomic scale, that oligocellulose adsorbs on the ice‐water interface, induces curvature‐driven freezing‐point depression through the Gibbs‐Thomson effect, and stabilizes dynamic quasi‐liquid domains that maintain ionic conductivity under freezing conditions. Benefiting from this molecular‐level mechanism, the symmetrical battery assembled with 10 mg mL^−1^ OC‐8 modified 1 m zinc trifluoromethanesulfonate electrolyte can maintain longer battery life, running steadily for over 1200 h at 1 mA cm^−2^/1 mAh cm^−2^. Furthermore, the assembled full Zn||NVO battery can operate stably for over 3000 cycles at −30°C and sustain a specific capacity of 122 mAh g^−1^, standing out among the reported sugar antifreeze electrolyte additives in recent years by its capacity and cycling performance. This work demonstrates a novel, bio‐inspired strategy for engineering high‐performance and durable aqueous electrolyte strategies for extreme low‐temperature energy storage.

## Supporting Information

The authors have cited additional references within the Supporting Information [39, 45, 46, 47, 48, 49, 50, 51, 52, 53, 54, 55, 56].

## Author Contributions

**Chong Gao**: Writing – review and editing, supervision, resources, funding acquisition, formal analysis. **Minmin Liang**: writing – review and editing, software, supervision, resources, project administration, funding acquisition. **Haoran Ma**: software, data curation, writing – original draft, investigation, visualization. **Zhiyuan He**: writing – review and editing, funding acquisition, resources, supervision, project administration, conceptualization. **Wei Li**: writing – review and editing, resources, validation, funding acquisition, conceptualization. **Cristina Carucci**: visualization, data curation, software, resources. **Kaiqi Li**: methodology, visualization, writing – review and editing. **Boya Yuan**: software, methodology, validation, data curation, formal analysis. **Dan Wang**: writing – review and editing, methodology, software, data curation, funding acquisition. **Sunghak Park**: conceptualization, methodology, visualization, software, data curation. **Jingxuan Pan**: conceptualization, methodology, validation, writing – review and editing, writing – original draft. **Zeyu Zhu**: writing – original draft, writing – review and editing, conceptualization, methodology, validation, investigation, data curation, visualization. **Xiaoting Chen**: writing – review and editing, conceptualization, methodology, resources, supervision, project administration.

## Conflicts of Interest

The authors declare no conflicts of interest.

## Supporting information




**Supporting File**: advs77291‐sup‐0001‐SuppMat.pdf.

## Data Availability

The data that support the findings of this study are available in the supplementary material of this article.
