# Supplementary material for: Bio‐Inspired Oligocellulose‐Regulated Ice‐Water Interfaces Enable Sustained Ion Transport in Frozen Aqueous Zinc Batteries
Source: Adv Sci (Weinh). 2026 Aug 24:e77291. Online ahead of print. doi: 10.1002/advs.77291 (PMC13502360; doi:10.1002/advs.77291)
Supplement: Supplementary file 1 [file ADVS-9999-e77291-s001.pdf]

# Supporting Information

## Bio-Inspired Oligocellulose-Regulated Ice-Water Interfaces Enable Sustained Ion Transport in Frozen Aqueous Zinc Batteries

Zeyu Zhu,[a],[e] Jingxuan Pan,[a],[e] Haoran Ma,[a],[e] Boya Yuan,[a],[e] Cristina Carucci,[b] Sunghak Park,[c] Chong Gao,[d] Minmin Liang,[a] Kaiqi Li,*[a] Wei Li, *[a],[d] Dan Wang,*[a],[d] Xiaoting Chen,*[a],[d] and Zhiyuan He*[a],[d]

[a] Z. Zhu, J. Pan, H. Ma, B. Yuan, Prof. M. Liang, Dr. K. Li, Prof. W. Li, Prof. D. Wang, Prof. X. Chen, Prof. Z. He

School of Materials Science and Engineering, Beijing Institute of Technology, Beijing 100081, P.R. China

E-mail: likq@bit.edu.cn; weili2021@bit.edu.cn; dan.wang@bit.edu.cn; xt.chen@bit.edu.cn; hezy@bit.edu.cn

[b] Cristina Carucci

Department of Chemical and Geological Sciences, University of Cagliari & Center for Colloid and Surface Science (CSGI), 09042 Monserrato, CA, Italy

[c] Sunghak Park

Department of Future Energy Engineering, Sungkyunkwan University, Suwon 16419, Republic of Korea

[d] Prof. C. Gao, Prof. W. Li, Prof. D. Wang, Prof. X. Chen, Prof. Z. He

Tangshan Research Institute, Beijing Institute of Technology, Beijing 100081, P.R. China

[e] Z. Z, J. P, H. M and B. Y contributed equally to this work.

## Table of Contents

1. Materials and Experimental Procedures....................................................................................S2

2. Supplementary Figures..............................................................................................................S5

3. References ...............................................................................................................................S21

## Experimental Procedures

### 1. Materials and Methods

### 1.1 Materials

Zinc trifluoromethanesulfonate (ZOTf, Macklin, 98%), oligocellulose (OC-6, OC-8, OC-15, OC-20, ITfiber, 99%), α-D-Glucose (Glucose, Aladdin, 96%), D-(-)-Arabinose (Arabinose, Aladdin, 98%), Sucrose (Aladdin, 99%), D-(+)-Trehalose Dihydrate (Trehalose, Macklin, 99%), Carboxymethyl cellulose (CMC, Aladdin, $M_w$ = 25000), Guar gum (Aladdin, 350-700 mPa·s), Carboxymethyl chitosan (CMCS, Macklin, Degree of substitution ≥ 80%), Lactobionic acid (Macklin, 97%), Sorbitol (Macklin, 98%), Xylitol (Aladdin, 98%), Ammonium vanadate ($NH_4VO_3$, Aladdin, 99%), N-Methylpyrrolidone (NMP, Aladdin, 99%), Super P, Polyvinylidene fluoride (PVDF), Commercial zinc foil (100 μm, 99.99%), Copper foil (50 μm, 99.99%), Titanium foil (15 μm, 99.99%). Milli-Q water was provided by a Millipore purification system.

### 1.2 Processing of electrodes

Polish the purchased commercial zinc and copper sheets with 1000 grit sandpaper until the surface is shiny, and then wash them with ultrasonic waves in isopropanol for 30 min. After ultrasonication, cut the foil into 12 mm diameter discs used for electrodes.

In addition, we synthesized ammonium intercalated vanadium pentoxide as a positive electrode based on previously reported literature for the preparation of Zn||$NH_4^+$-$V_2O_5$ full cells.[1] In brief, we placed 0.1 g of $NH_4VO_3$ in a high-temperature tube furnace and heated it at 300 °C with a heating speed of 10 °C $min^{-1}$ for 2 h in argon atmosphere to attain the $NH_4^+$-$V_2O_5$ powder.

In order to prepare the $NH_4^+$-$V_2O_5$ electrode, we mix the $NH_4^+$-$V_2O_5$, Super P and PVDF with a mass ratio of 7:2:1 in a mortar (The excess of $NH_4^+$-$V_2O_5$ powder is about 3%). After grinding for 15 minutes,

add 260 μL of NMP to the mortar and continue grinding for another 15 minutes. Then, coat the obtained slurry onto washed titanium foil and place it overnight in an 80 °C oven. Finally, cut the obtained electrode into a disc with a diameter of 12 millimeters for future use. The mass loading of $NH_4^+$-$V_2O_5$ was about 0.75 mg $cm^{-2}$ to 1.25 mg $cm^{-2}$.

**1.3 Preparation of saccharide-modified electrolyte**

Add the synthesized OC-8 and selected sugars (including glucose, arabinose, sucrose, trehalose, CMC, guar gum, CMCS, lactobionic acid, sorbitol, xylitol) at a concentration of 10 mg $mL^{-1}$ to a 1 M ZOTf solution, and sonicate for 30 min to obtain the electrolyte for battery testing.

**1.4 Characterization**

Mass spectrometry (MS) of OC-8 was performed on AUTOFLEX SPEED (Bruker, Germany). Nuclear magnetic resonance hydrogen spectrum ($^1$H-NMR) of OC-8 was performed on AVANCE NEO 400 WB (Bruker, Germany). X-ray diffraction (XRD) measurement of OC-8 was performed in a D8 Advance instrument (AXS-Bruker, Germany) with Cu Kα irradiation (λ = 1.54 Å). Fourier transform infrared (FT-IR) analysis of OC-8 was performed on Thermo Scientific Nicolet iS50 (Nicolet, America). Confocal experiment was performed in a fluorescence microscope (LSM980, Zeiss, Germany). Differential scanning calorimetry (DSC) to test the freezing point of electrolyte was performed on Mettler Toledo Calorimeter (Mettler Toledo, Switzerland).

**1.5 Ice recrystallization experiments**

We conducted experiments on ice recrystallization based on previously reported splat cooling methods.[2] The experimental setup includes a cold stage (Instec, America) for freezing and temperature control, a polarizing microscope for observation (LV100ND, Japan), and a camera for shooting (Nikon Y-TV55, Japan). In terms of the experimental procedure, a droplet of tested solutions (about 10 μL) was dropped onto the cover glass precooled to −60 °C on the cryo-stage from a height of 1.5 m to form a thin solid ice film. Then we raise the temperature to −8 °C at a rate of 10 °C $min^{-1}$ and anneal for 40 min. Three experimental runs were performed for every sample. Then we statistically analyze and calculate the error bars for the size of ice crystals formed in the experiment.

**1.6 Nanoliter Osmometer Experiments**

We use a YASN Nanoliter Osmometer (YASN, England) to test and a high-spped camera (Phantom v2012, America) to observe the growth of individual ice crystals. After calibration, we add a drop of silicone oil onto a specially designed glass slide, and then use a microsyringe to add 0.2 μL tested solution into the oil. During the experimental process, we froze the dispersion and let it slowly warm to its melting temperature.[3] Once a small (~15-20 μm in diameter) single ice crystal appeared, it was kept for about 20 s and the temperature was recorded as the melting temperature ($T_m$). Then the temperature was decreased to the desired value ($T_f$). Supercooling temperature ($\Delta T$) is obtained by subtracting $T_f$ from $T_m$. Experiments were repeated for three times and calculate the ice crystal growth rate obtained by dividing the difference in ice crystal diameter by time.

**1.7 Electrochemical Experiments**

Cyclic voltammetry (CV) experiments and electrochemical impedance spectroscopy (EIS) tests are performed on an electrochemical work station (Metrohm Autolab PGSTAT302N). Among them, EIS was measured to calculate the ionic conductivity of the electrolyte by assembling the Cu||Cu battery at a frequency of 0.1-100 kHz with the following formula:

$$\sigma = \frac{l}{R \cdot S}$$

where R denotes the resistance tested through the EIS, *l* is the distance between the cathode and anode, and S is the contact area between the electrodes and the electrolyte.

In terms of assembling the cells, all coin batteries are prepared in standard CR2032 cells. All low-temperature experiments were operated in a freezer facility with stationary temperature and humidity (SEM-64PF-70, −70-150 °C). The battery is assembled in sequence from a negative electrode protective case, spring washer, stainless steel washer, negative electrode, separator film (Glass fiber, GF/A, Whatman), positive electrode, and positive electrode protective case. In all battery tests, the processed Zn disc is used as the negative electrode. Zn disc, Cu disc and NVO cathode are used as the positive electrodes to assemble symmetrical batteries, asymmetrical batteries, and full batteries for testing, respectively.

**1.8 Molecular Dynamic (MD) Simulation**

Molecular dynamics simulations were performed using GROMACS software.[4] The initial ice

geometric structures were constructed using GenIce2.[5] The water-ice system consists of a 7.364 × 5.992 × 1.565 nm ice crystal and 12,124 free water molecules, which serve to inhibit downward ice growth. To enforce this constraint, a layer of water molecules beneath the initial ice seed was immobilized using the *freezegrps* directive in GROMACS. The OC-8 was placed above the ice layer. The period boundary condition (PBC) was applied in all directions for the simulations. The long-range electrostatic interaction was described by the Particle-Mesh Ewald (PME) method[6] with the cutoff of 1.0 nm. The cutoff of the Van der Waals interaction was also set to 1.0 nm. All the simulation systems were energy-minimized with a criterion of the maximum force < 1000 kJ/mol/nm and then were equilibrated under constant NPT ensemble for 1 ns with Parrinello-Rahman barostat at 1 bar pressure. After equilibration, an additional 100 ns NPT simulation was carried out by using a Nose-Hoover thermostat at 243K with a coupling time of 0.1 ps. The Glycam06 force field[7] and TIP4P/Ice[8] model were employed for OC-8 and water in all MD simulations. The snapshots of the systems are processed using VMD software.[9] Ice-like water molecules were identified by using the CHILL+ algorithm, accessed via the open-source OVITO Python package.[10-11] Four independent simulation systems were constructed to isolate the effects of OC-8 and $Zn^{2+}$, respectively: pure water-ice, water-ice with OC-8, water-ice with $ZnCl_2$, and water-ice with both OC-8 and $ZnCl_2$. For each system, a single 100 ns production trajectory was generated. All time-series data presented in Figure 3 are derived from these representative trajectories.

**Supplementary Figures**

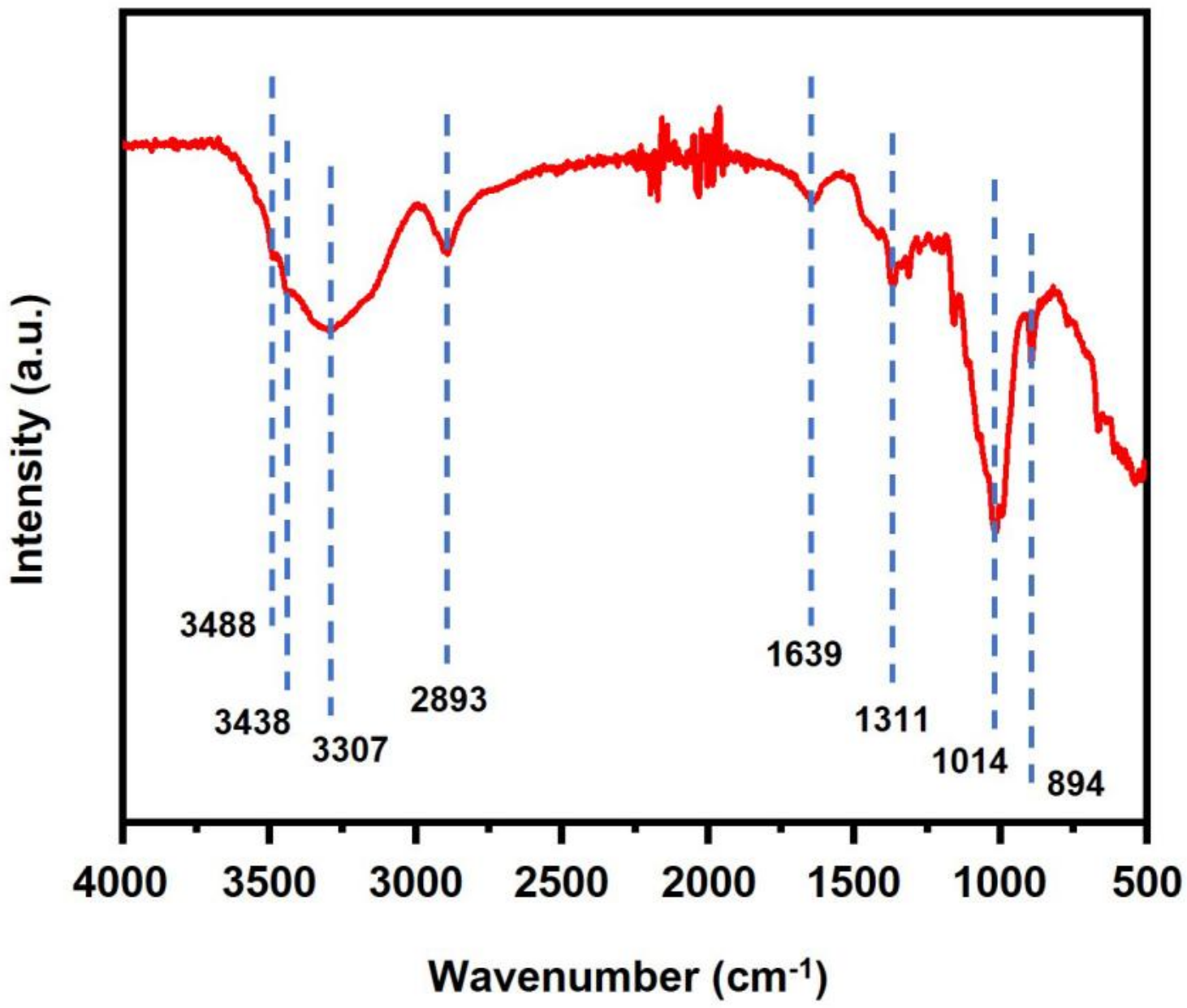


**Figure S1.** FT-IR spectra of OC-8. Among them, bands in the range from 3500 to 3000 $cm^{-1}$ are assigned to hydrogen-bonded stretching of OH. The peaks at 2893 $cm^{-1}$, 1639 $cm^{-1}$ are attributed to CH stretching vibration, OH bending vibration respectively. The bands at 1311 $cm^{-1}$ and 1014 $cm^{-1}$ are the C-H deformation vibration and C-O-C stretching vibration, respectively. The C-H bending vibration is showed at 894 $cm^{-1}$.[12]

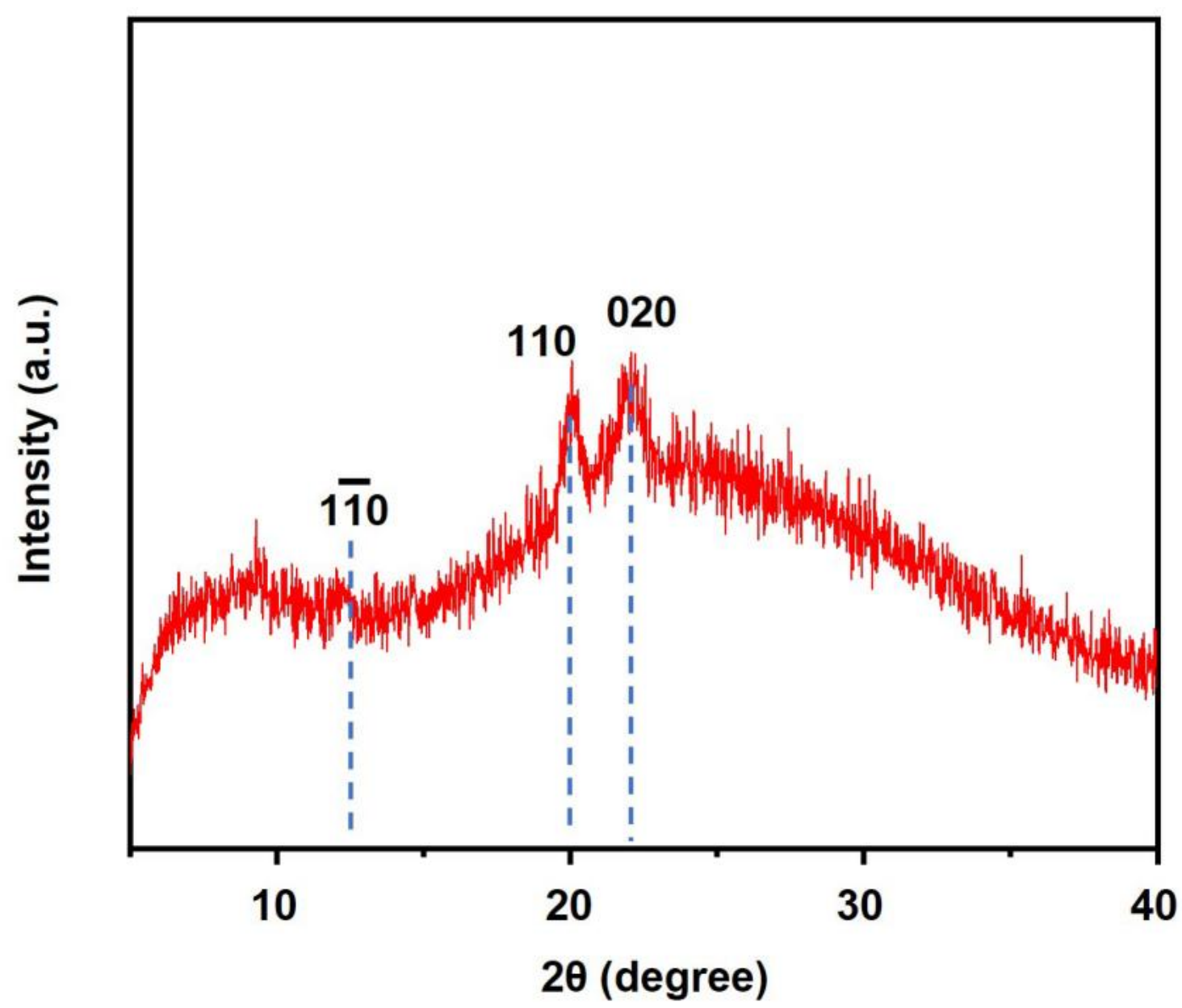


**Figure S2.** XRD pattern of OC-8. Among them, the crystal plane peak in the crystalline state is consistent with the previously reported literature.[12]

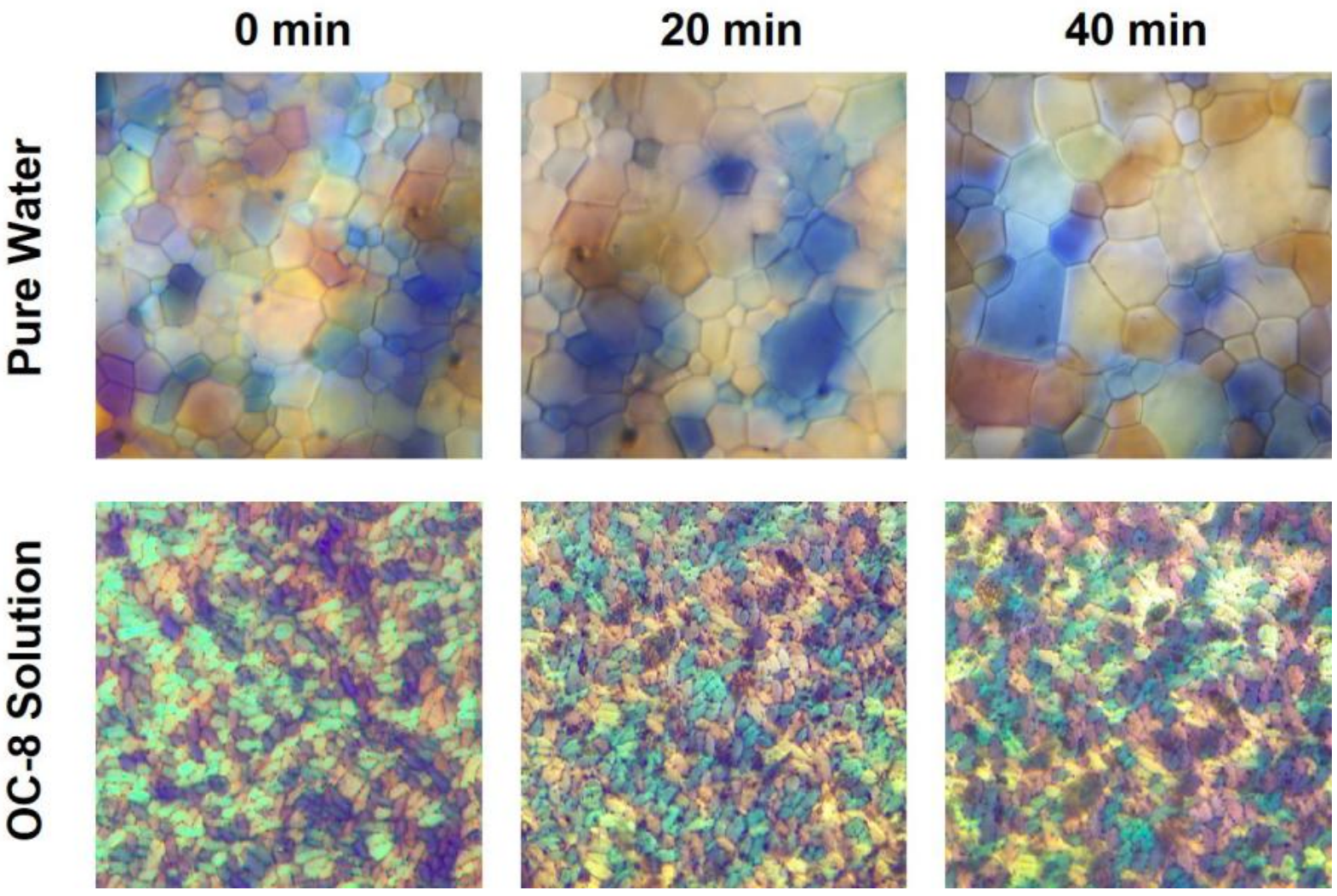


**Figure S3.** Microscopic images of ice recrystallization after 0, 20, and 40 minutes annealing at −8 °C.

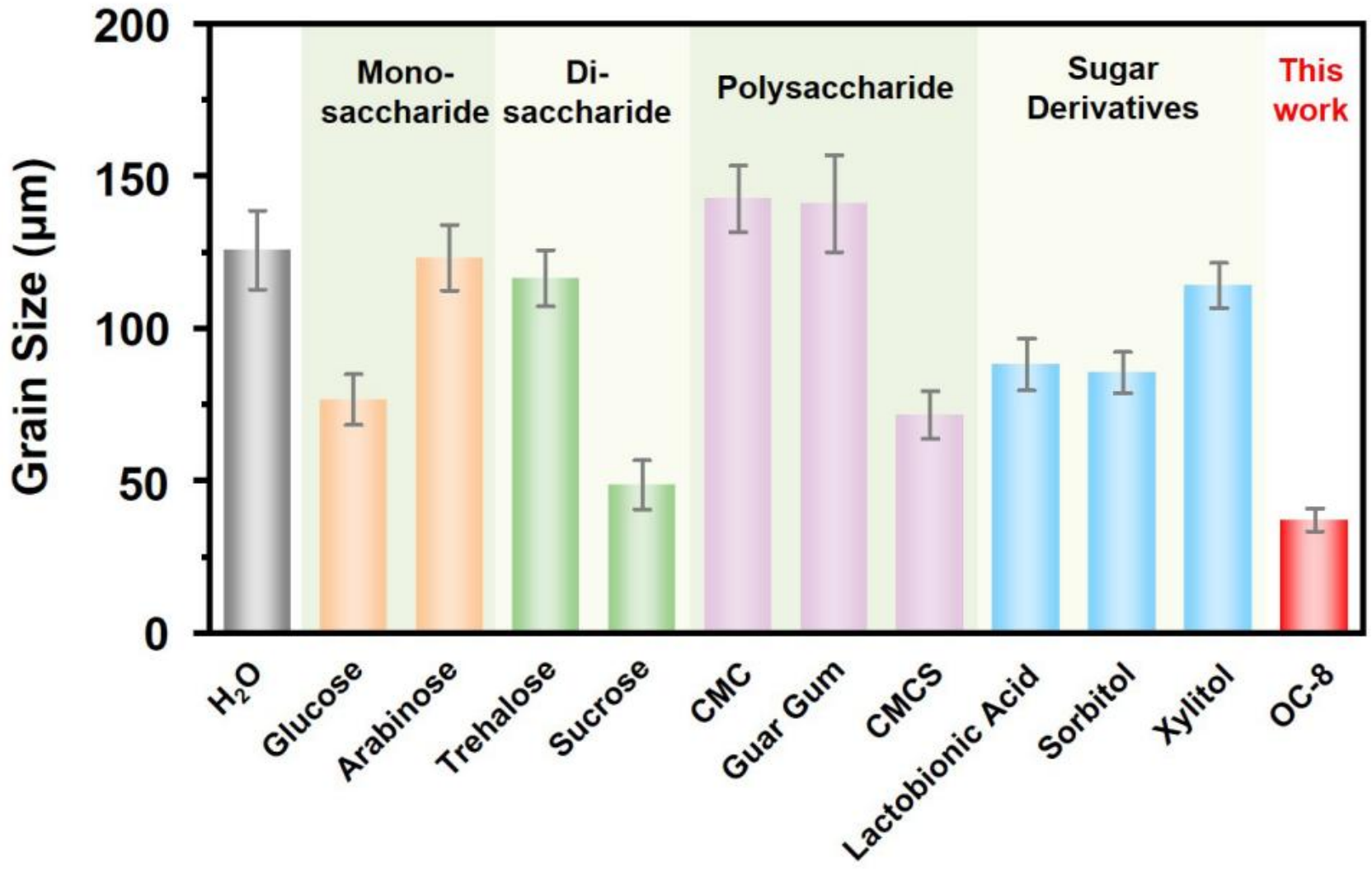


**Figure S4.** Grain size of ice crystals in aqueous solution with different kinds of saccharide at the same concentration of 10 mg mL$^{-1}$. The results demonstrate that OC-8 has extraordinary capability on inhibiting ice recrystallization and formation.

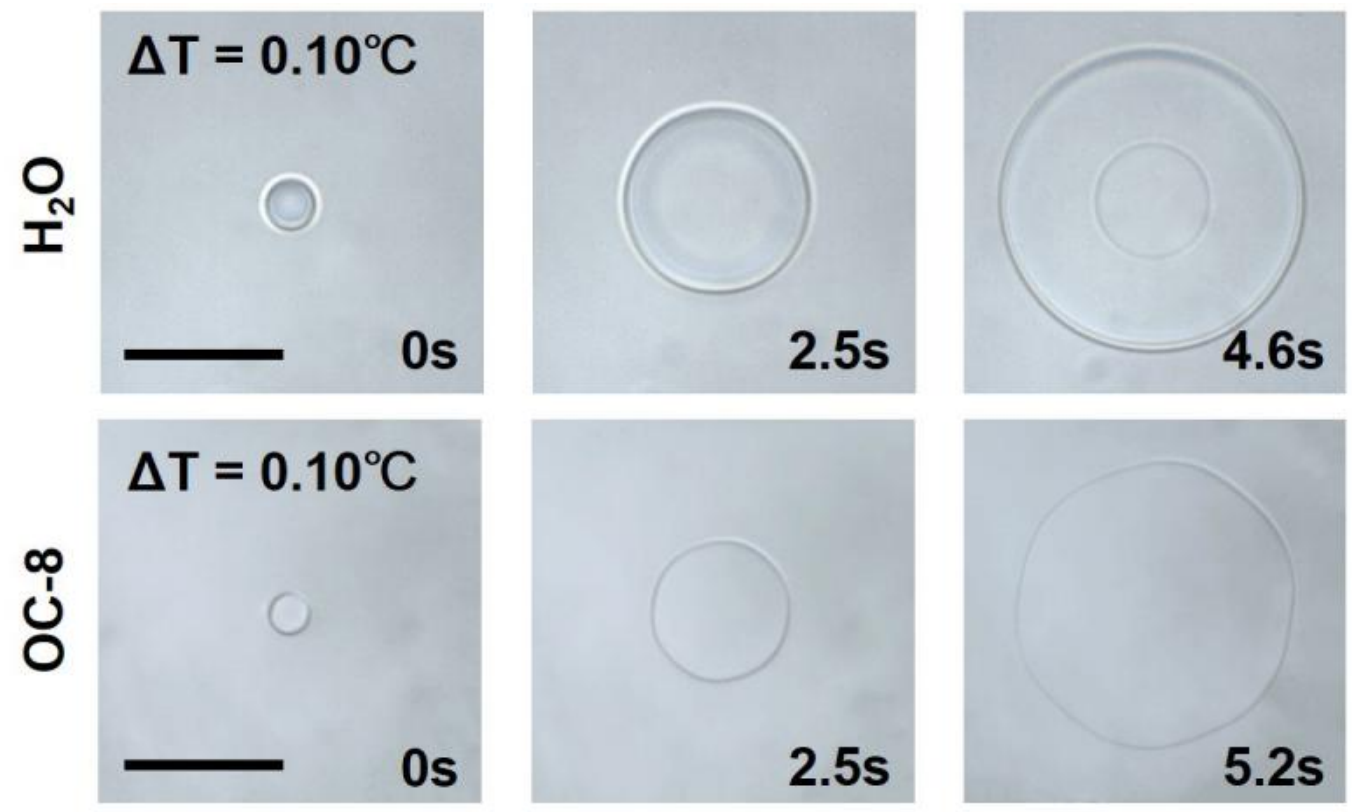


**Figure S5.** Images of single ice crystal growing in pure water and 10 mg $mL^{-1}$ OC-8 aqueous solution at a super cooling temperature (ΔT) of 0.10 °C. Scale bar: 100 μm.

Despite OC-8 does not have a particularly significant inhibitory effect on the growth of individual ice crystals, it can still modify the morphology of ice crystals into hexagons.

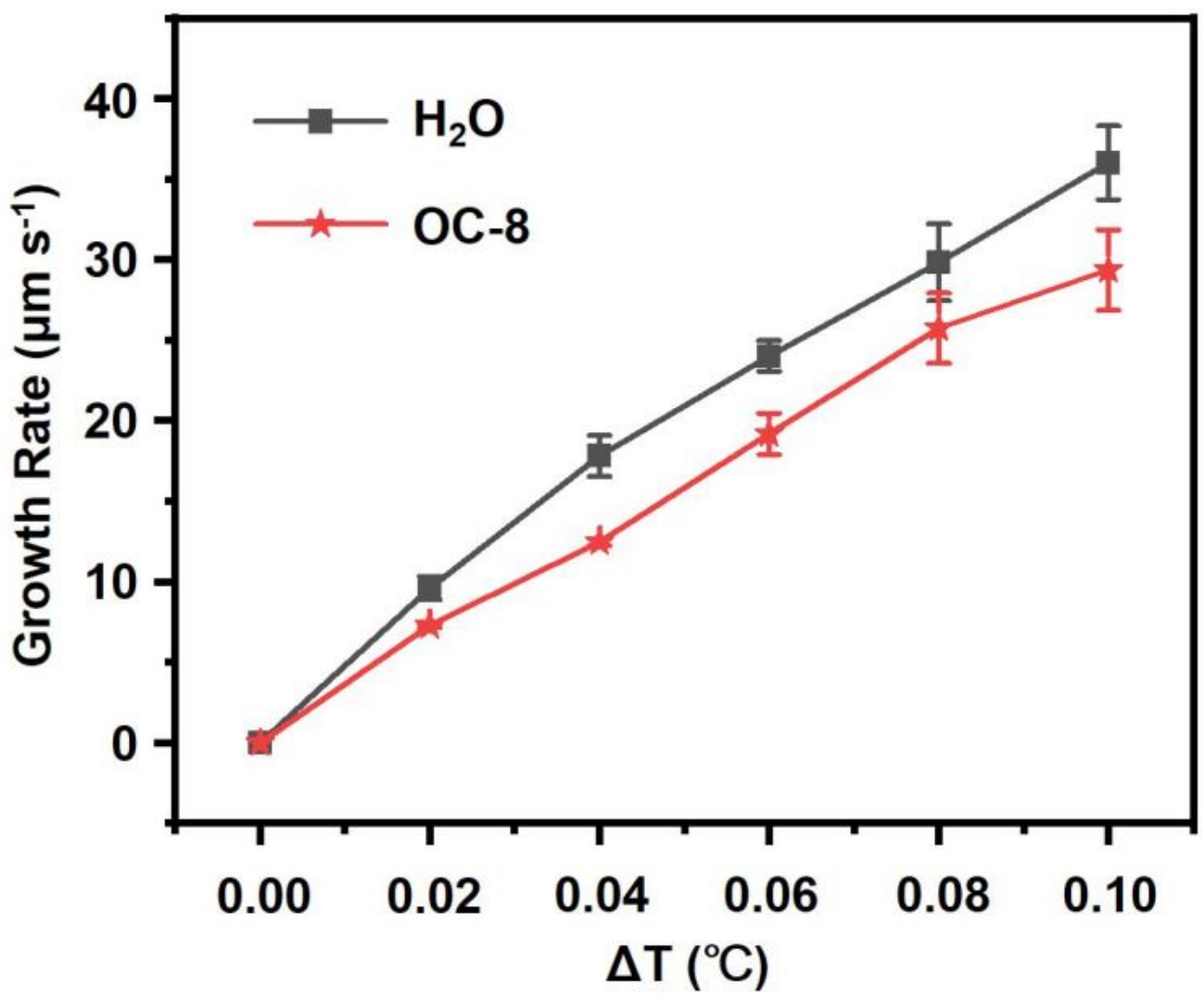


**Figure S6.** The growth rate of single ice crystal in pure water and 10 mg mL$^{-1}$ OC-8 aqueous solution under different ΔTs.

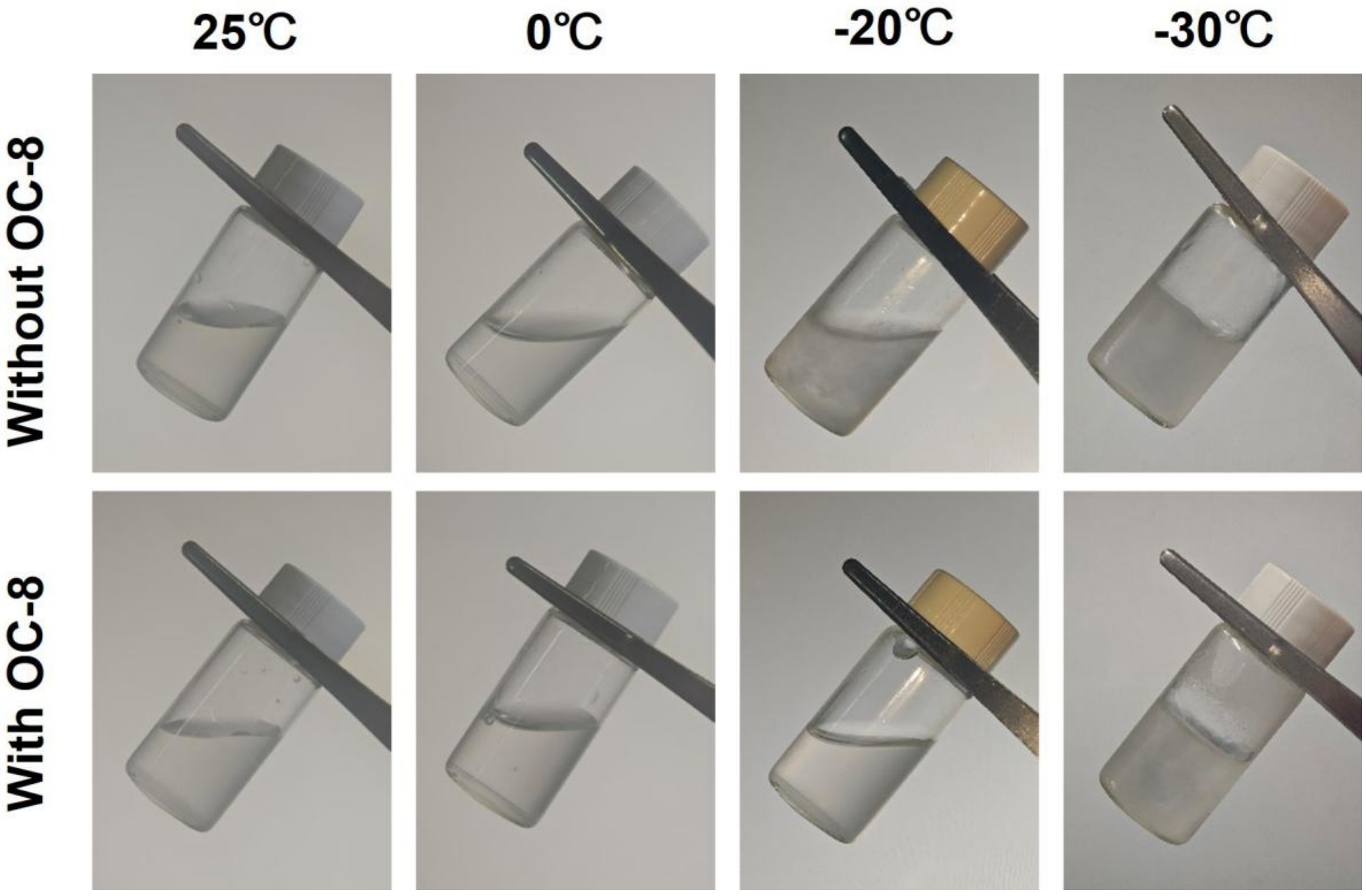


**Figure S7.** Macroscopic state images of electrolyte before and after adding OC-8 at different temperatures ranging from 25 °C to −30 °C.

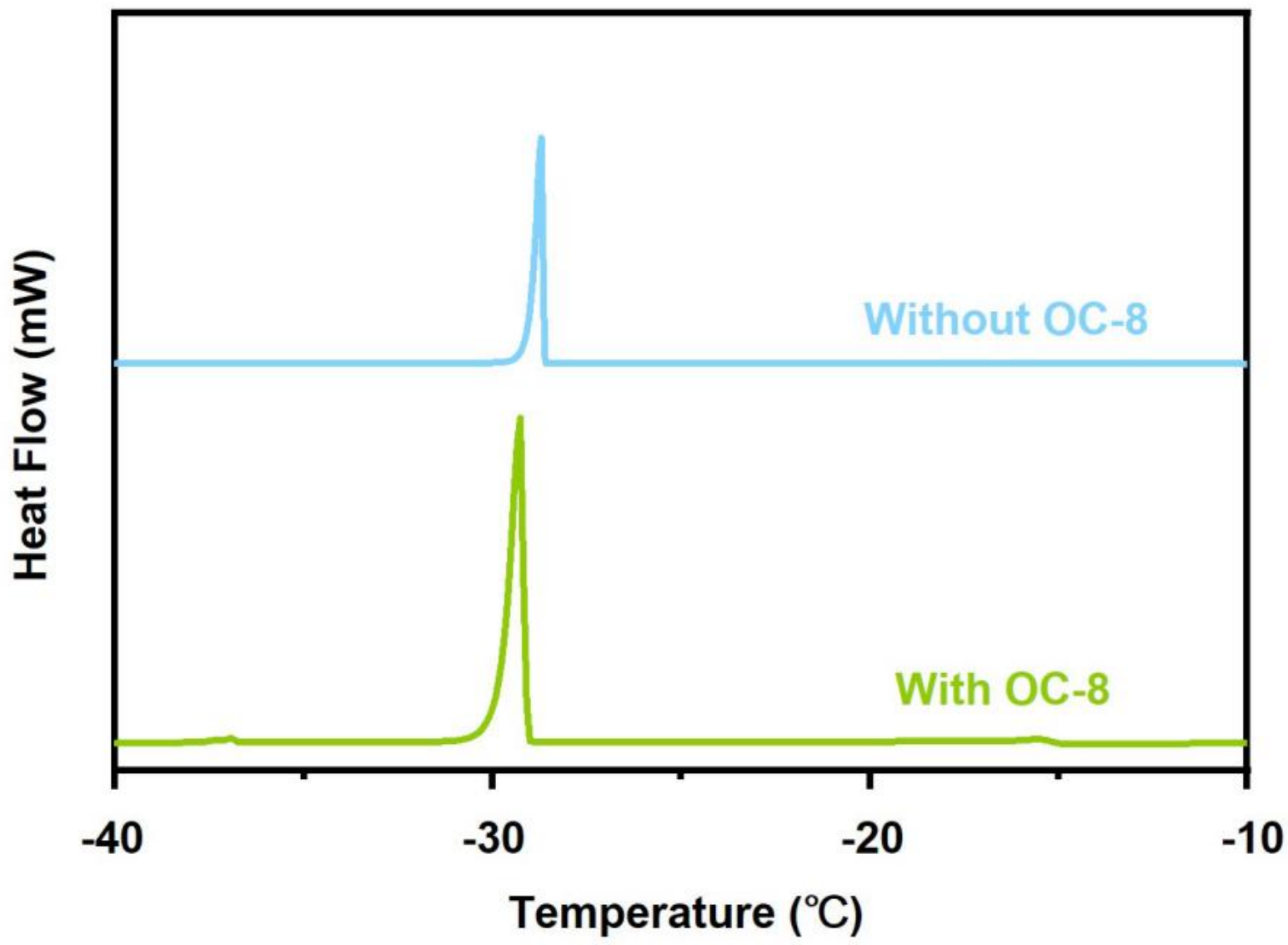


**Figure S8.** DSC results of electrolyte with and without the addition of OC-8.

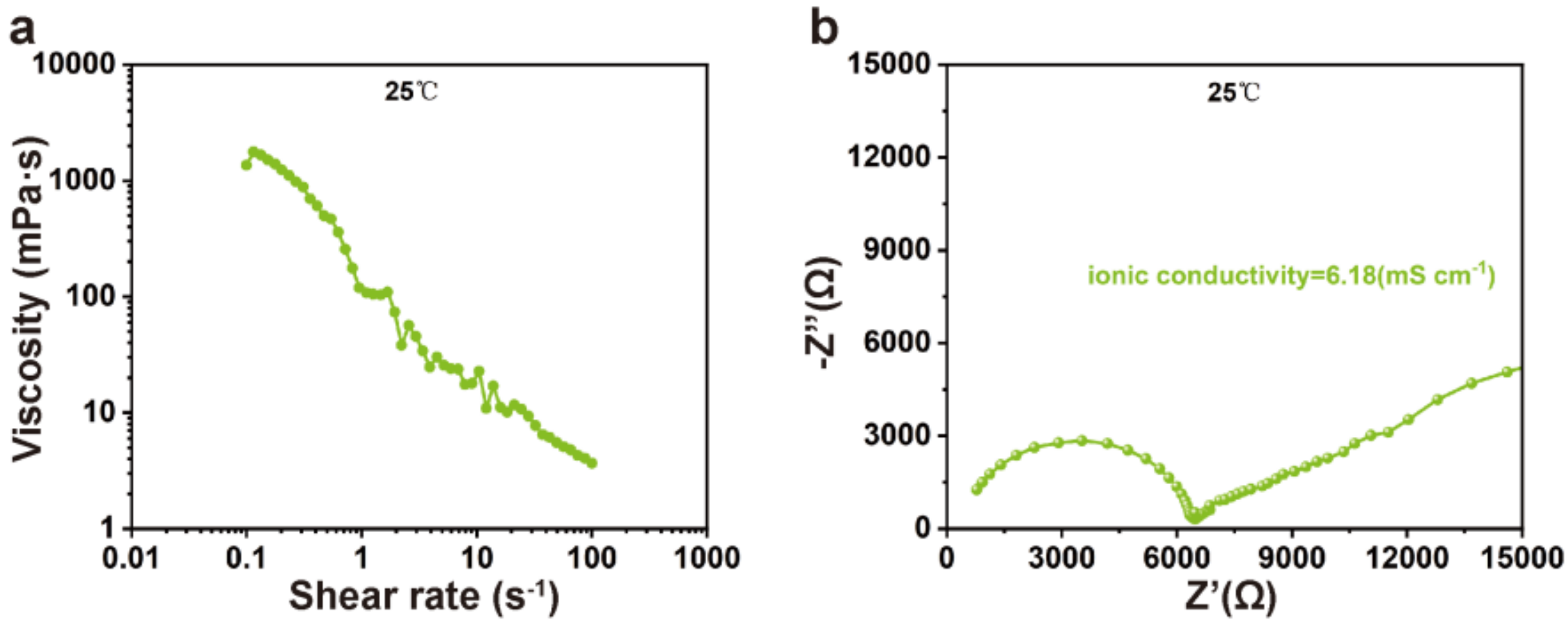


**Figure S9.** Rheological and ion-transport properties of the 10 mg mL$^{-1}$ OC-8 aqueous solution at 25 °C. a) Apparent viscosity as a function of shear rate, showing pronounced shear-thinning behavior. b) Nyquist plot used to determine the ionic conductivity. The calculated ionic conductivity is 6.18 mS cm$^{-1}$.

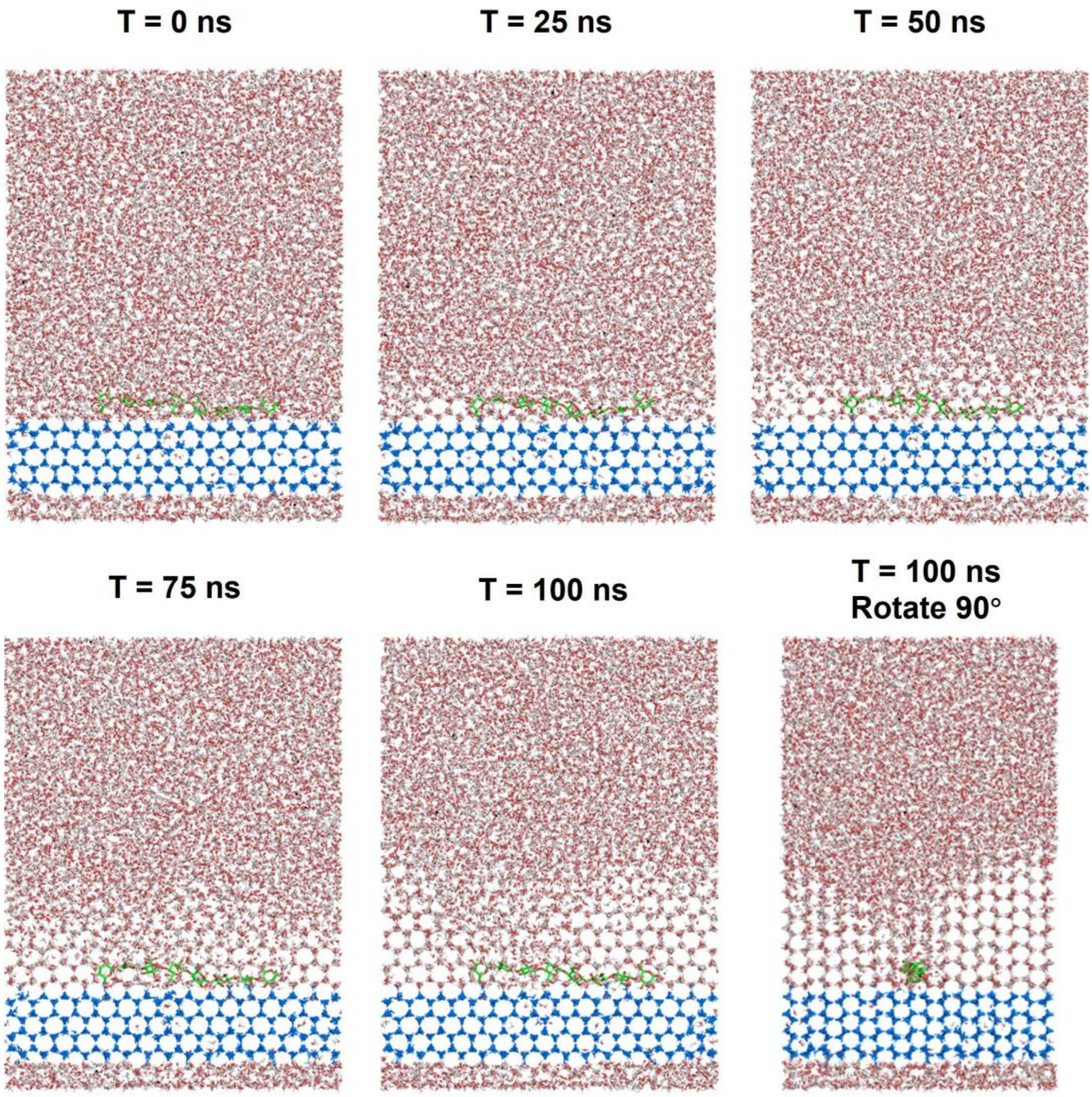


**Figure S10.** Molecular dynamics simulations (MD) over a 100-ns timescale at −30 ℃ reveal that the OC-8 (green chain) effectively inhibit ice growth. Representative MD snapshots at 0, 25, 50, 75, and 100 ns, along with the 100 ns structure viewed after a 90° rotation around the ice growth direction. Carbon, hydrogen and oxygen atoms are shown in green, white, and red respectively. The initial ice seed was shown in blue.

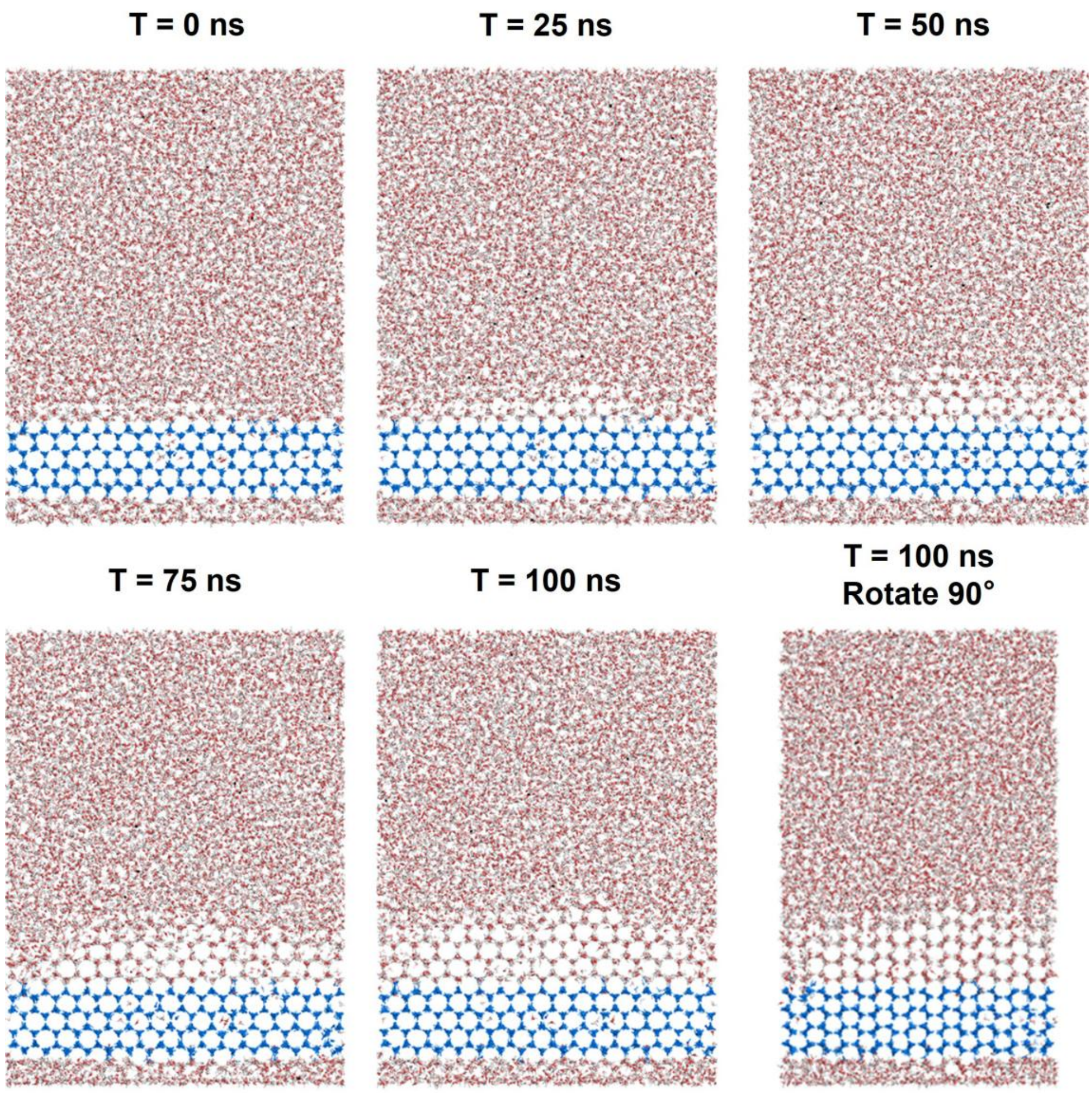


**Figure S11.** Molecular MD snapshots of water-ice system at −30 ℃, used as the control group in this study.

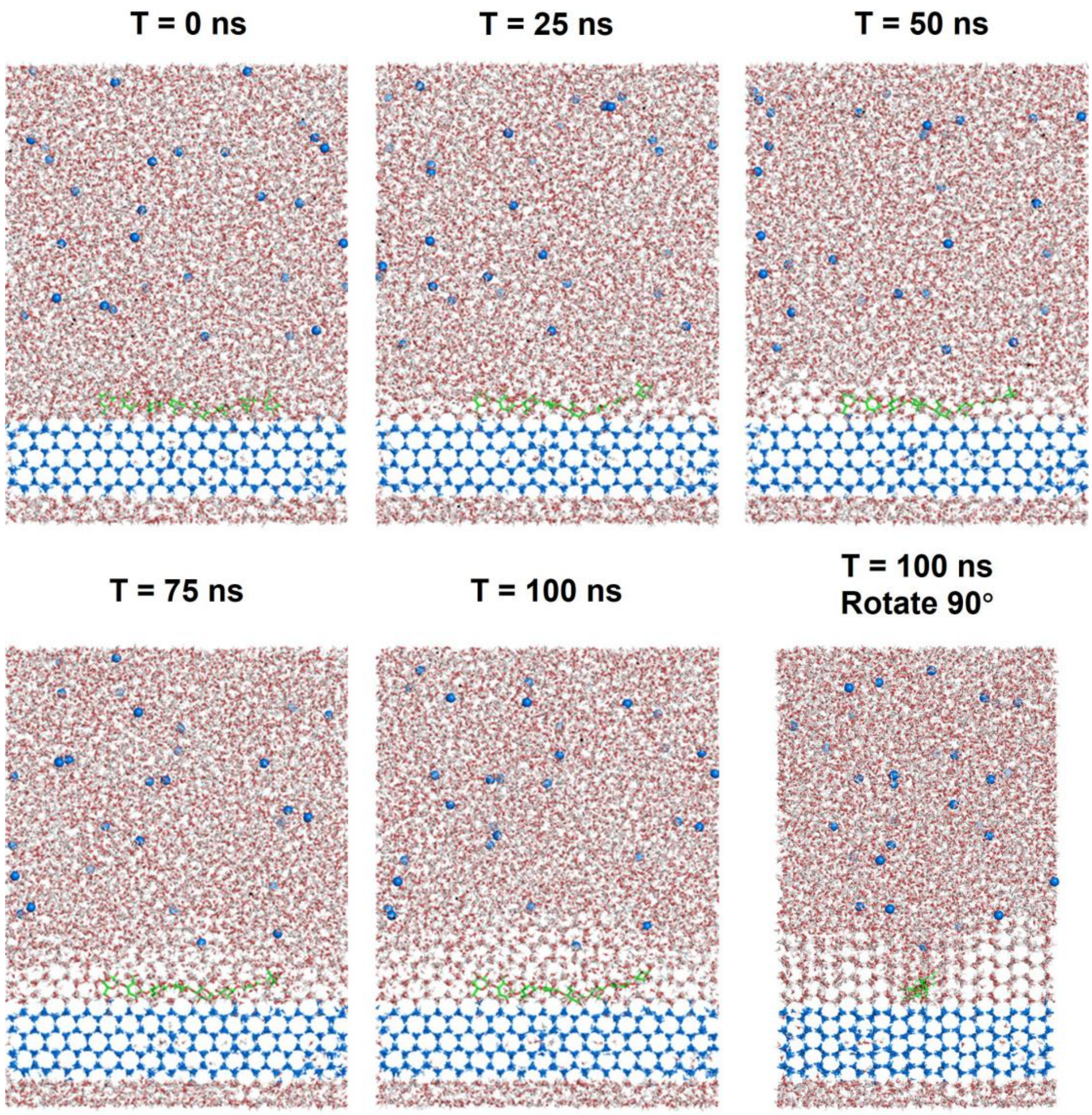


**Figure S12.** $ZnCl_2$ was used in the molecular dynamics (MD) simulations to investigate the ion transport behavior in such a system. This substitution was made to avoid the significant structural and volumetric complexity introduced by the bulky triflate ($OTf^-$) anions present in ZOTf, which was used as the zinc electrolyte in experimental procedures, on the simulation system, necessitating a simpler alternative like $ZnCl_2$ to streamline the process. It should be noted that the inclusion of ions in the MD simulations aims to explore ion transport mechanisms rather than to exactly replicate the experimental electrolyte composition or concentration. Additionally, high-concentration $ZnCl_2$ has demonstrated effectiveness in forming low-temperature aqueous zinc-ion batteries, though its ice-inhibition capability is limited at lower concentrations.[13] This figure presents an MD snapshots with the addition of 30 $ZnCl_2$ molecules. Carbon, hydrogen, oxygen, and zinc atoms are shown in green, white, red, and blue respectively.

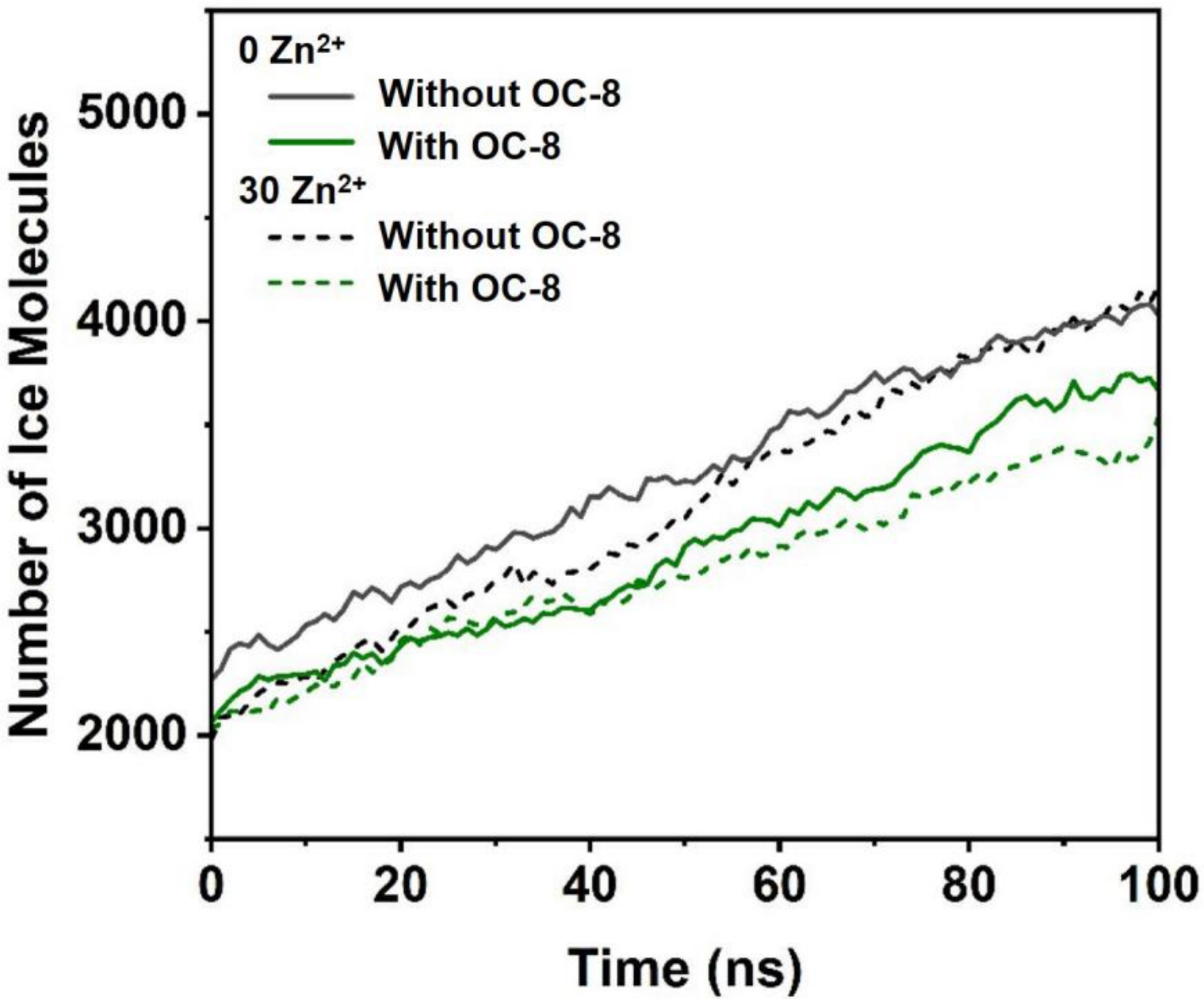


**Figure S13.** Inhibition of hexagonal ice growth of OC-8. Time evolution of the number of water molecules identified as hexagonal ice in MD systems with and without OC-8 at 0 and 30 $Zn^{2+}$ ions added. The analysis was performed by the CHILL+ algorithm. Lines styles distinguish between the two $Zn^{2+}$ concentration (solid for 0 $Zn^{2+}$, dashed for 30 $Zn^{2+}$), while colors differentiate the presence (green) or absence (black) of OC-8.

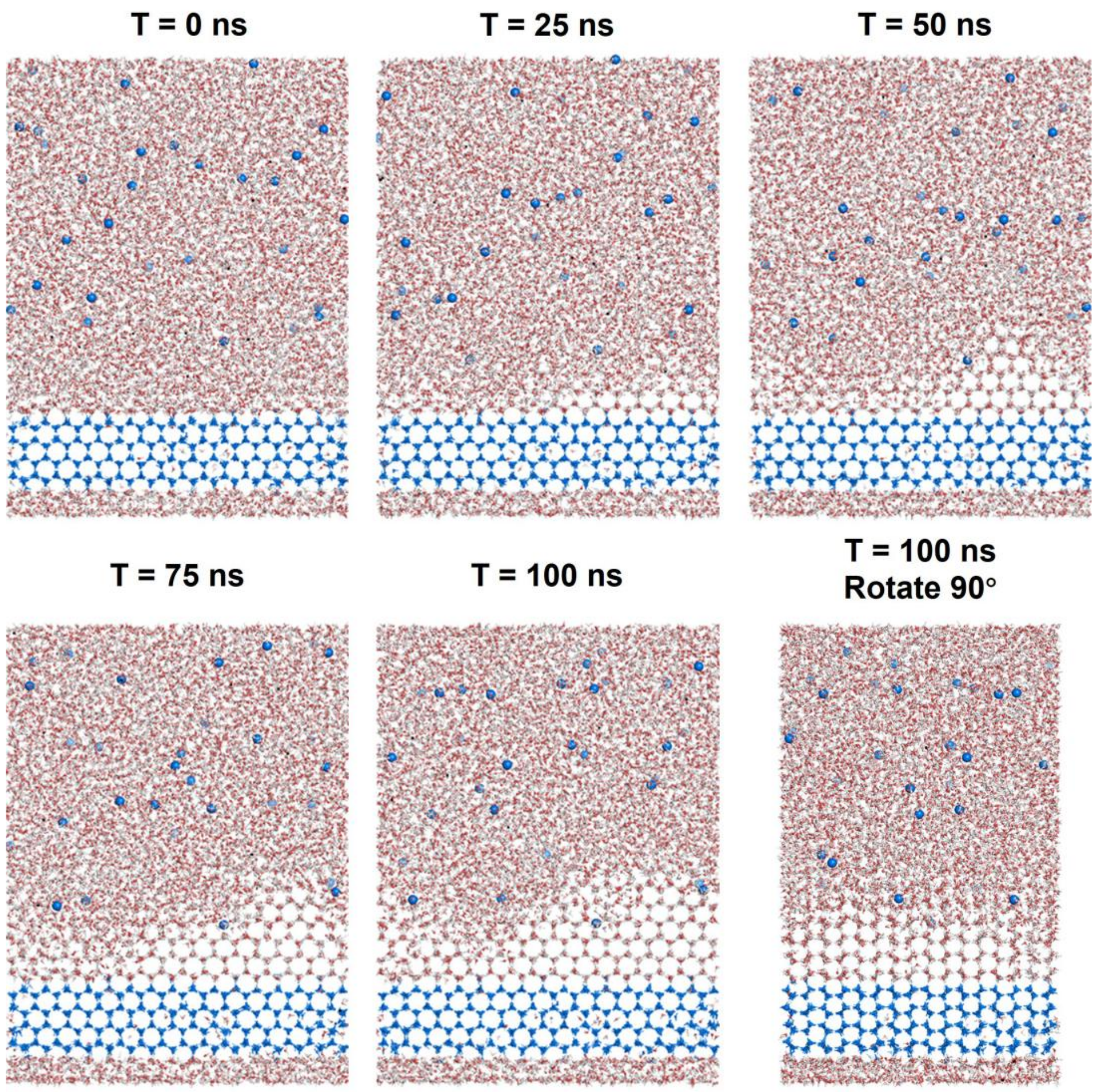


**Figure S14.** Water-ice box added 30 $ZnCl_2$ system MD snapshots at −30 °C.

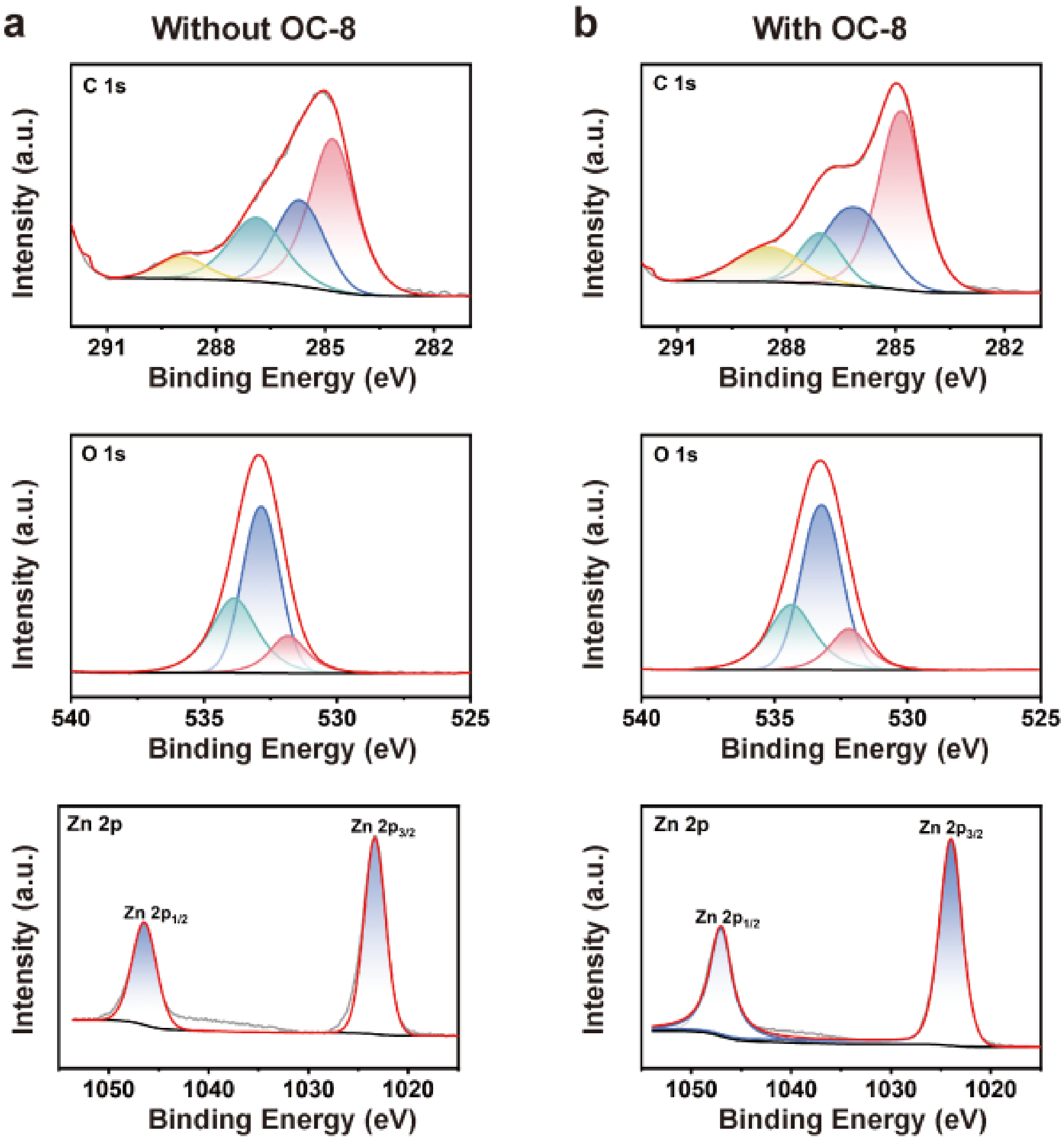


**Figure S15.** High-resolution XPS spectra of freeze-dried 1 M ZOTf electrolytes without and with 10 mg mL$^{-1}$ OC-8. a) C 1s, O 1s, and Zn 2p spectra of the ZOTf electrolyte. b) Corresponding spectra of the ZOTf-OC-8 electrolyte.

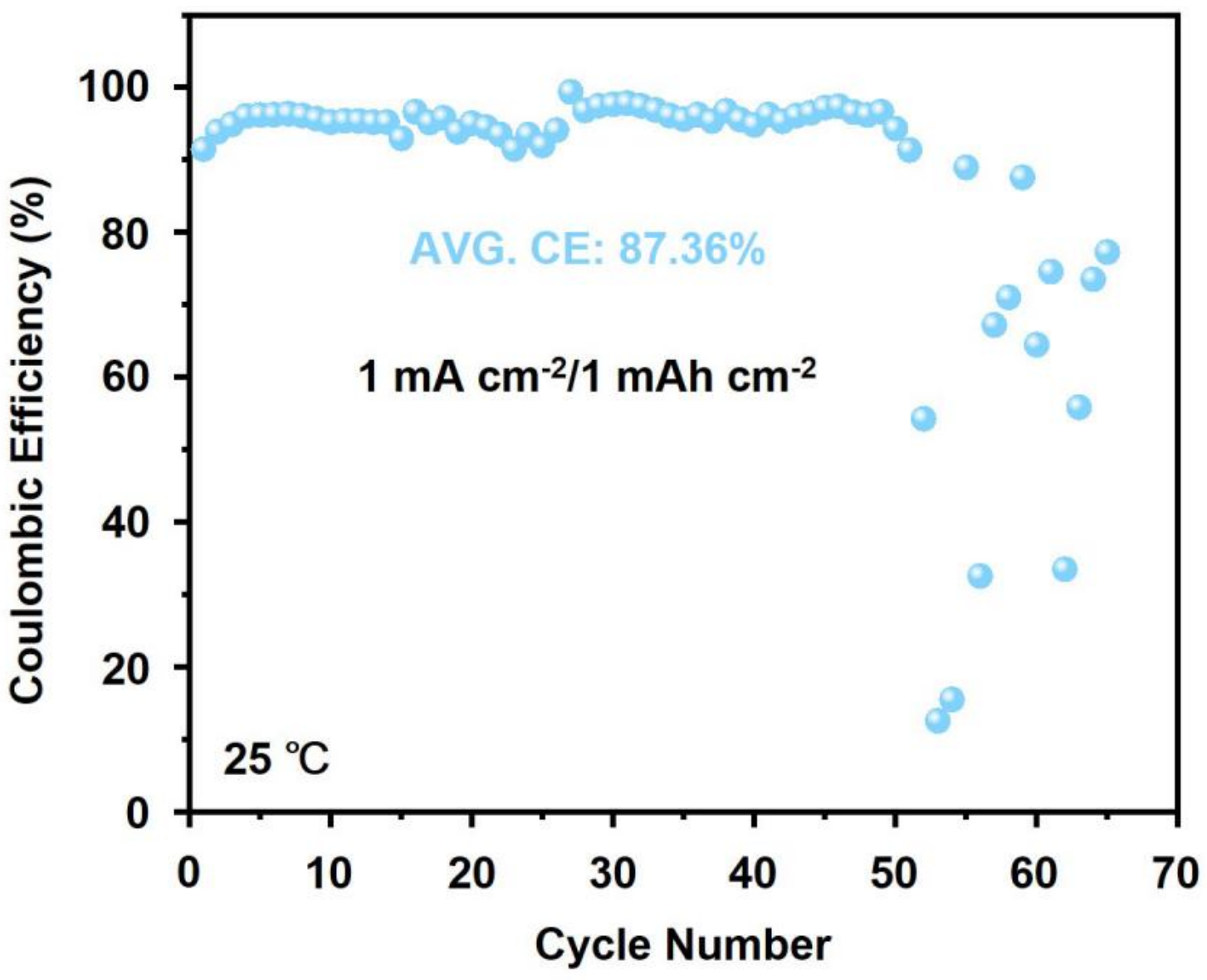


**Figure S16.** Operation results of Zn||Cu asymmetric battery assembled with blank electrolyte. The battery quickly fails after running for less than 70 cycles.

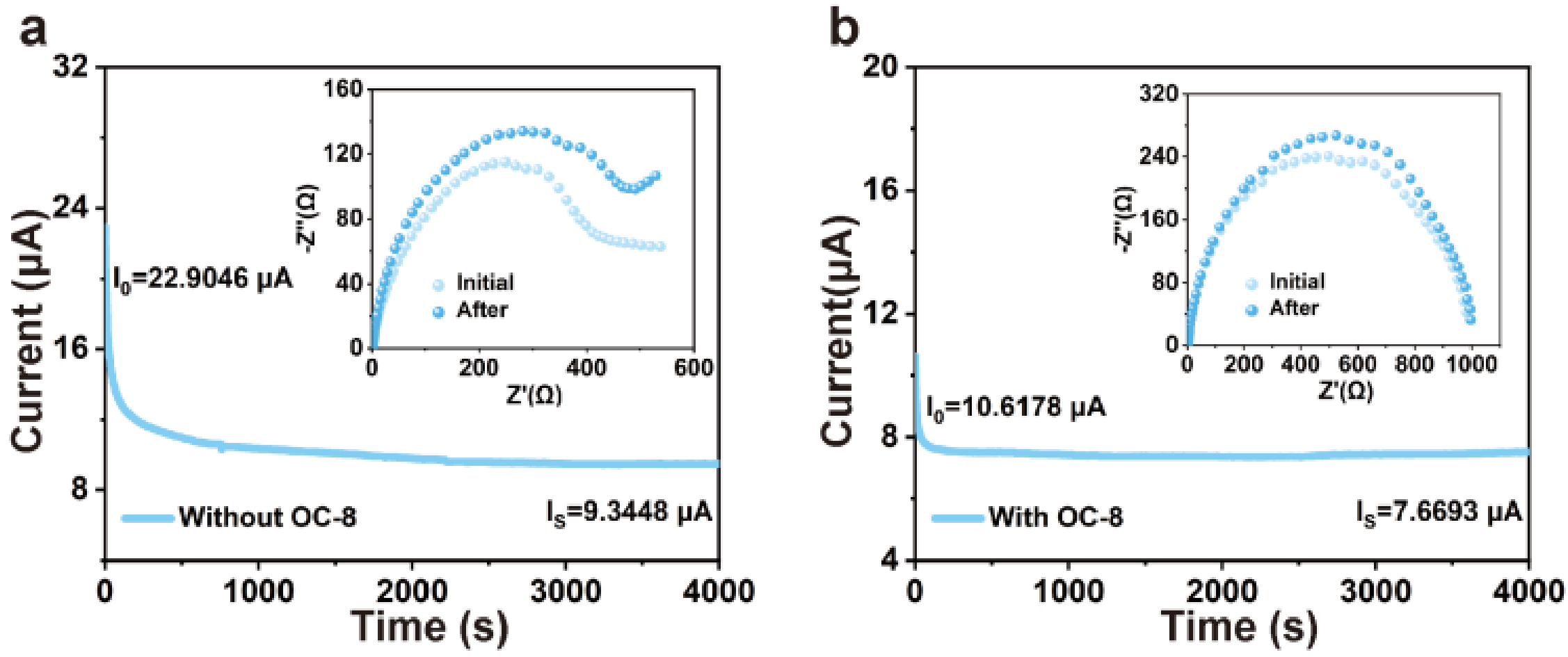


**Figure S17.** $Zn^{2+}$ transference-number measurements of a) ZOTf and b) ZOTf-OC-8 electrolytes at 25 °C. The main panels show chronoamperometric polarization curves of Zn||Zn symmetric cells under a DC voltage of 10 mV, and the insets show the corresponding Nyquist plots before and after polarization. The calculated $Zn^{2+}$ transference numbers are 0.07 and 0.24, respectively.

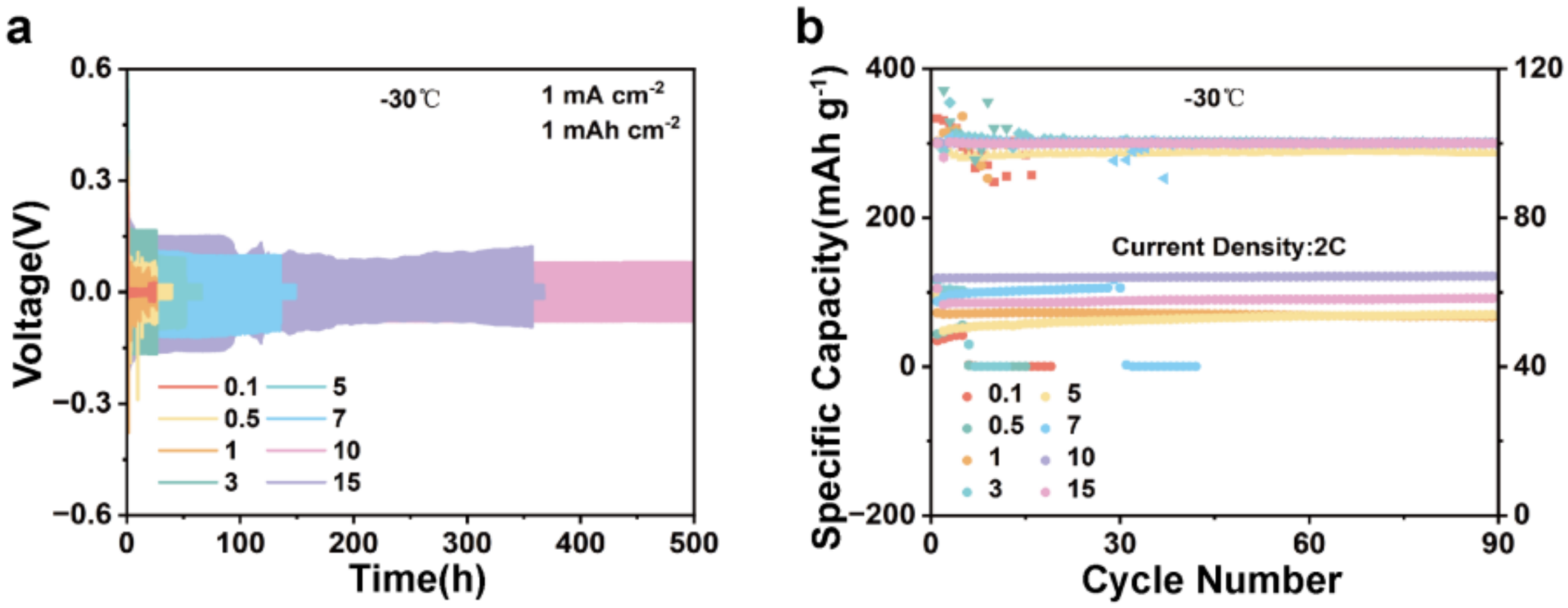


**Figure S18.** Concentration-dependent electrochemical performance of OC-8-modified ZOTf electrolytes. a) Long-term cycling performance of Zn||Zn symmetric batteries using ZOTf electrolytes containing different concentrations of OC-8 under −30 °C. b) Cycling performance of Zn||NVO full batteries using electrolytes containing different concentrations of OC-8 at −30 °C. The results indicate that 10 mg $mL^{-1}$ OC-8 delivers the best overall electrochemical performance.

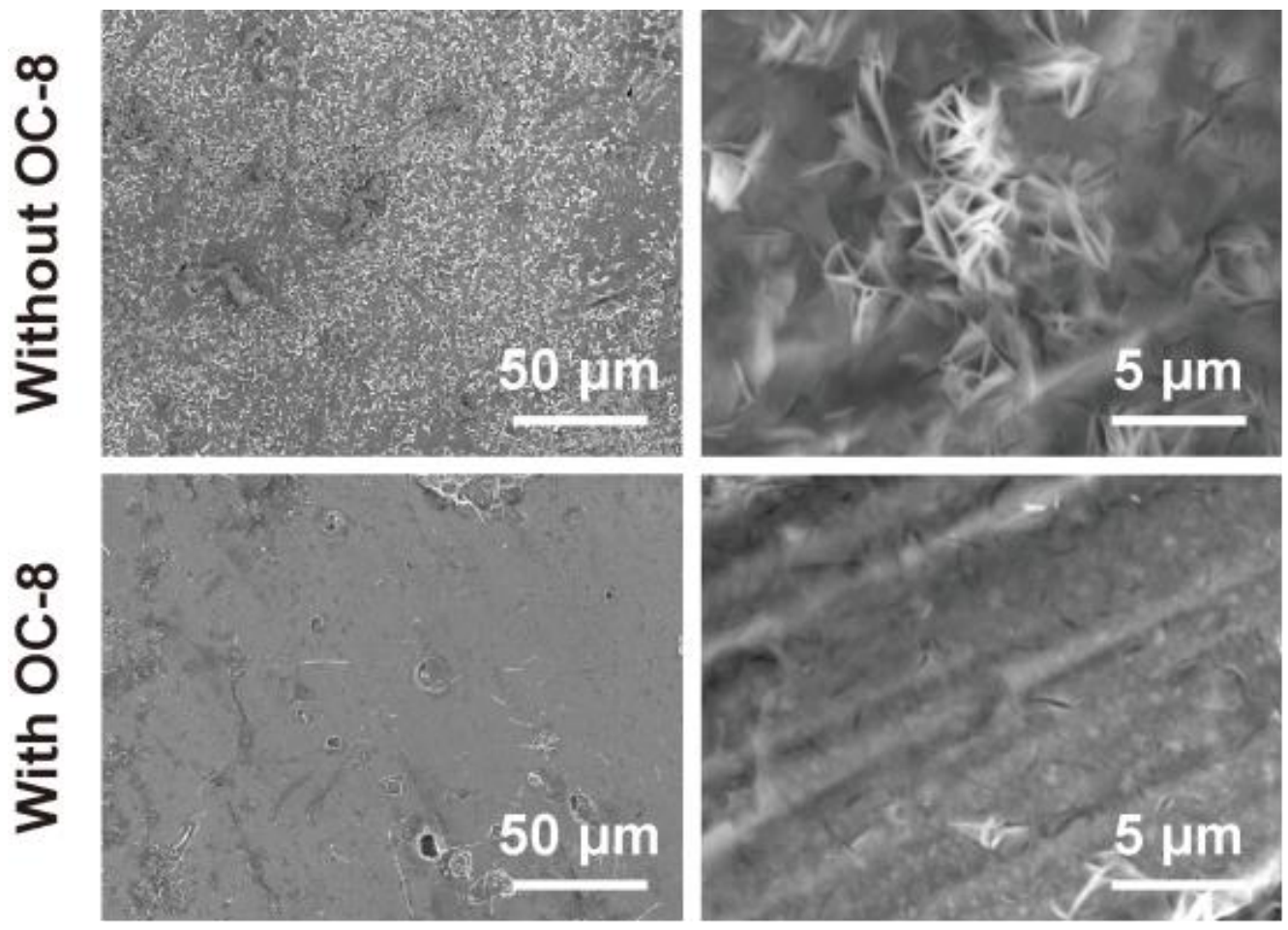


**Figure S19.** SEM images of Cu electrodes after 50 cycles in Zn||Cu cells at −30 °C using electrolytes with and without OC-8.

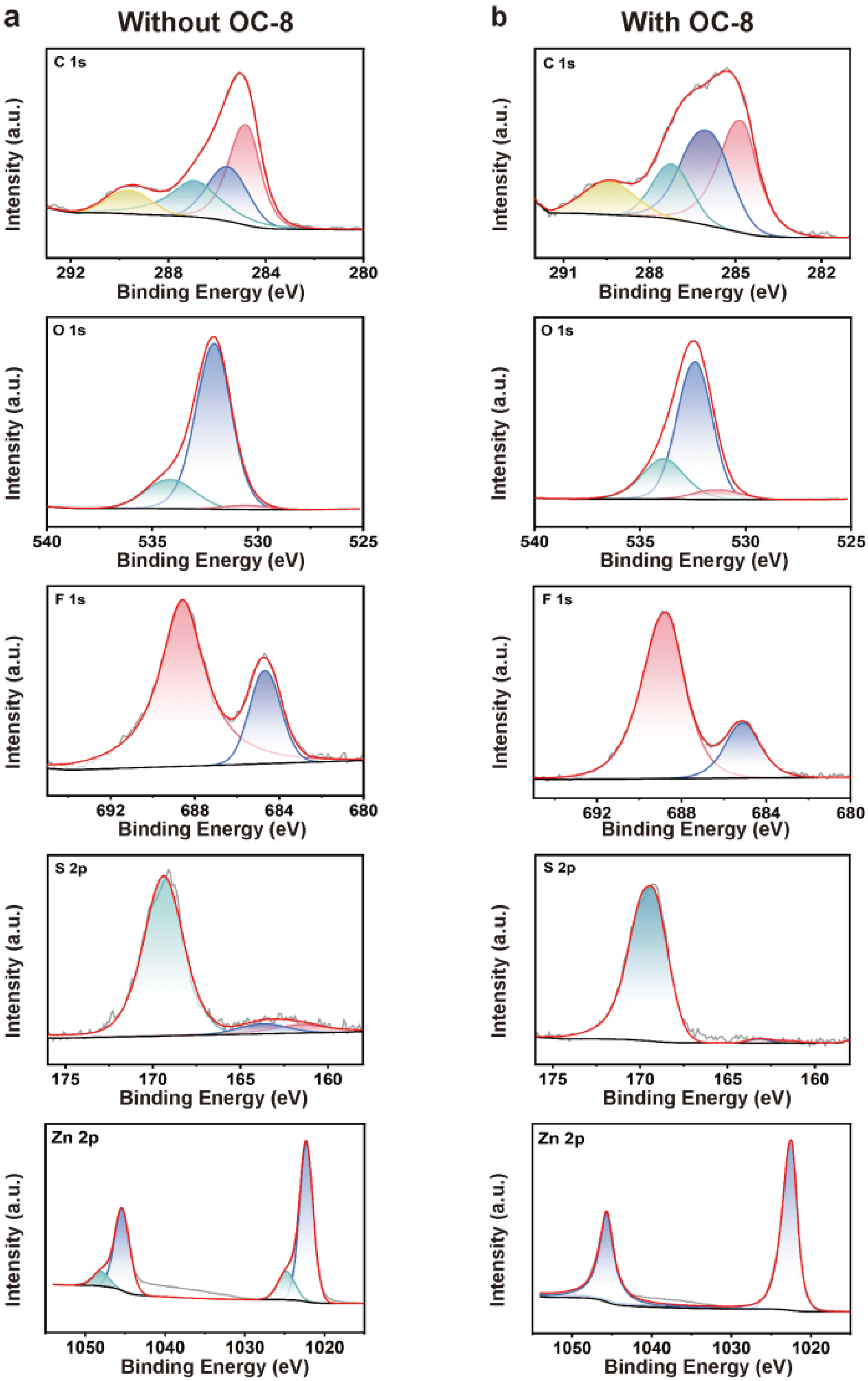


**Figure S20.** High-resolution XPS spectra of Zn electrodes retrieved from Zn||Zn symmetric cells after 50 cycles at −30 °C. a) C 1s, O 1s, F 1s, S 2p, and Zn 2p spectra of the Zn electrode cycled in the ZOTf electrolyte. b) Corresponding spectra of the Zn electrode cycled in the ZOTf-OC-8 electrolyte.

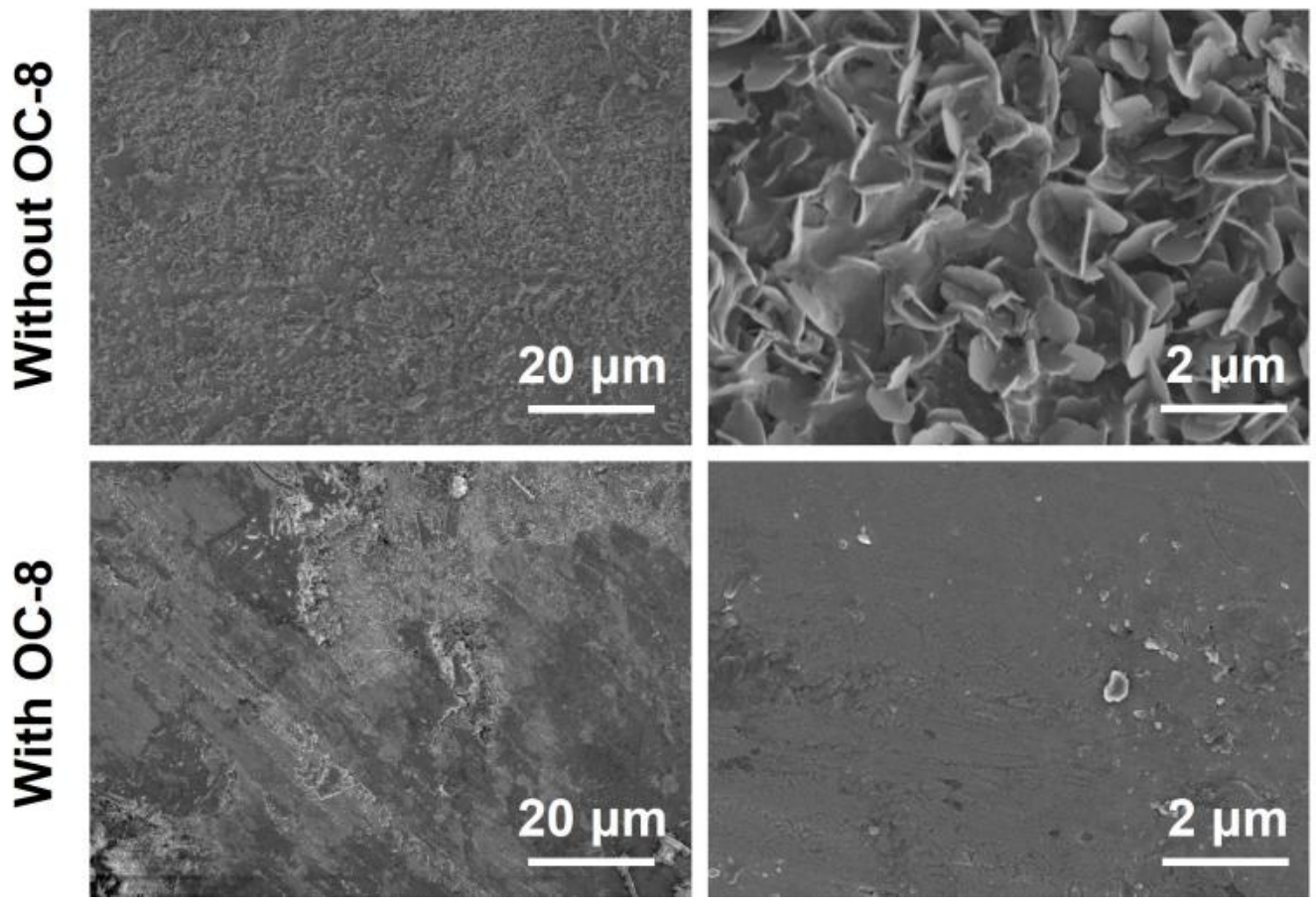


**Figure S21.** SEM images of the zinc negative electrode surface after 50 cycles of Zn||Zn symmetric battery operation before and after adding OC-8 at −30 °C.

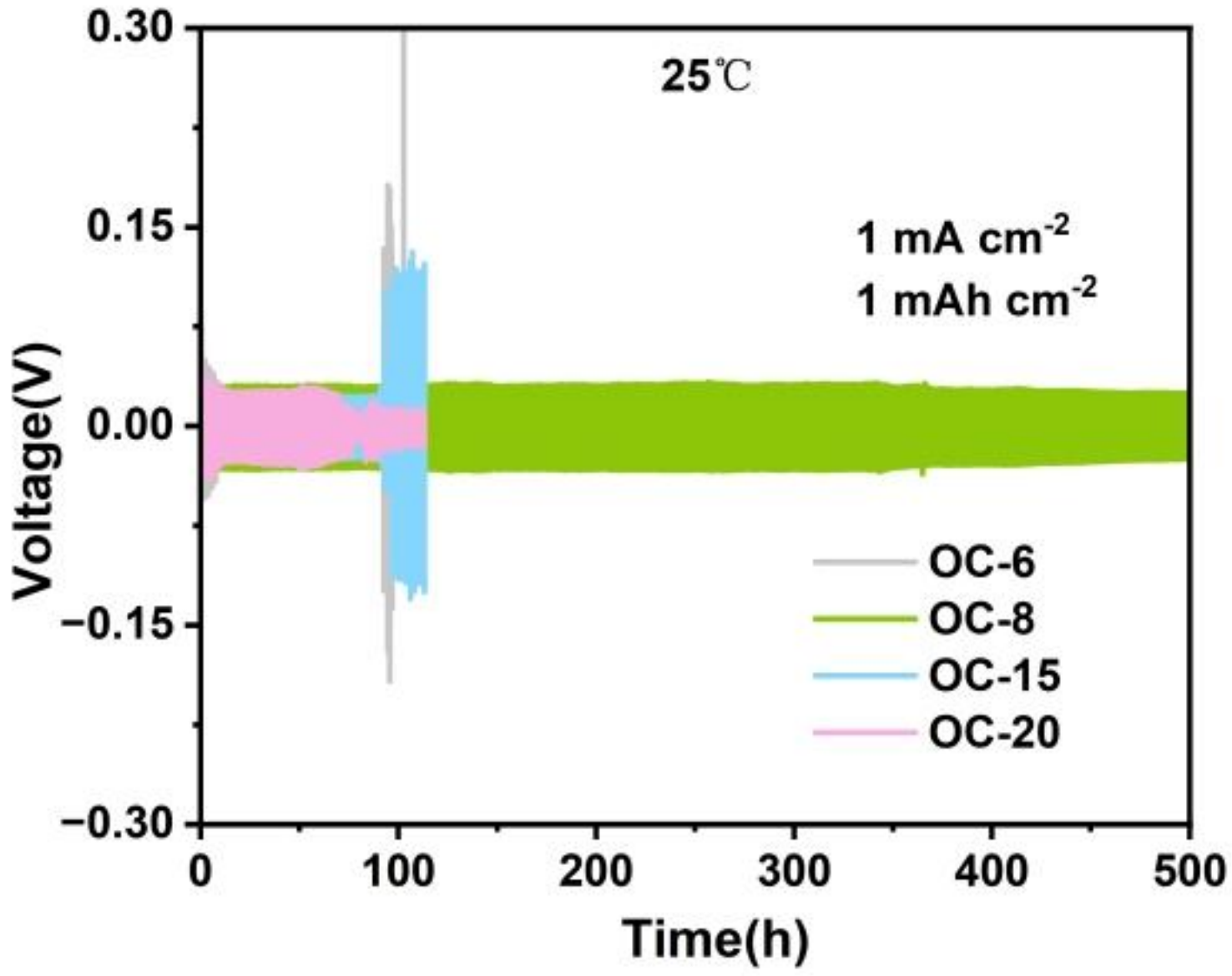


**Figure S22.** Room-temperature cycling performance of Zn||Zn symmetric cells using electrolytes containing OC-6, OC-8, OC-15, and OC-20 at 10 mg $mL^{-1}$. The OC-8-containing electrolyte shows the best overall cycling stability.

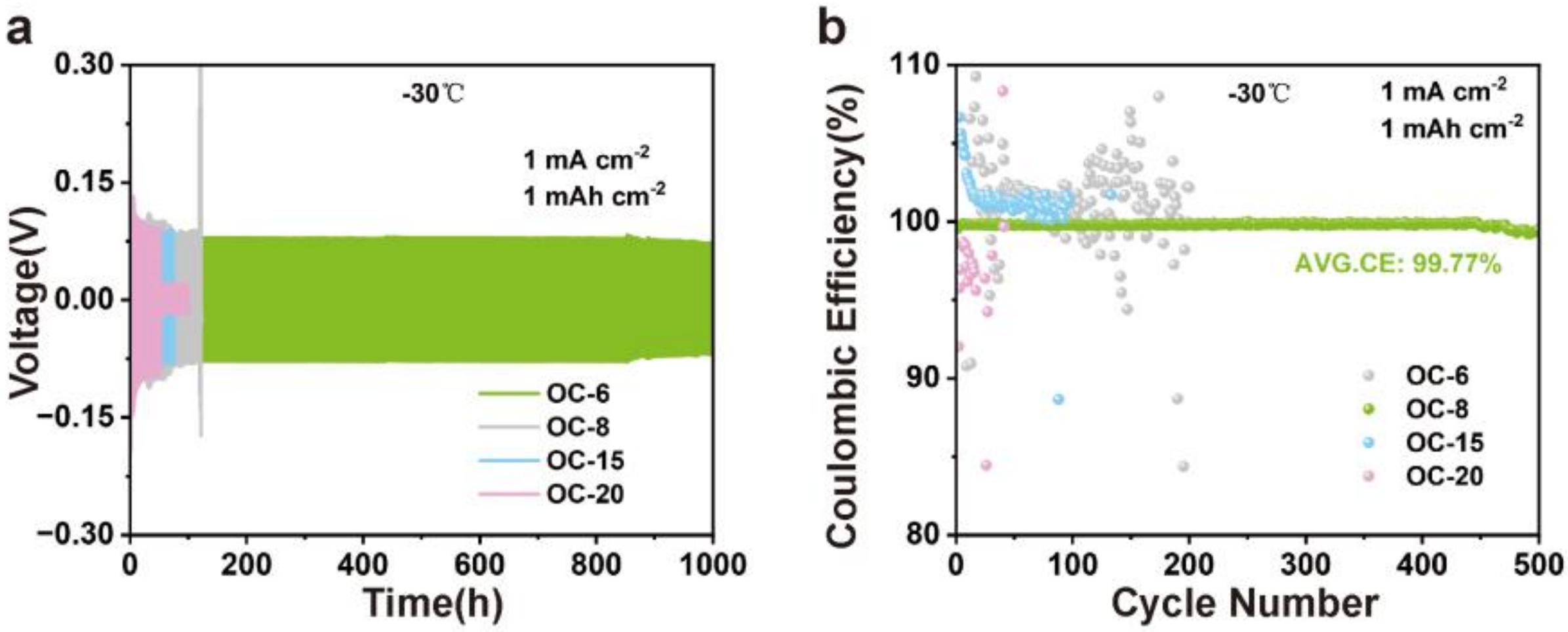


**Figure S23.** Electrochemical comparison of oligocellulose samples with different average degrees of polymerization at 10 mg $mL^{-1}$. a) Low-temperature cycling performance of Zn||Zn symmetric cells. b) Low-temperature electrochemical performance of Zn||Cu cells. The OC-8-containing electrolyte shows the best overall performance among the tested samples.

## Reference


[1] Y. X. Wang, S. Q. Wei, Z. H. Qi, S. M. Chen, K. F. Zhu, H. H. Ding, Y. Y. Cao, Q. Zhou, C. D. Wang, P. J. Zhang, X. Guo, X. Y. Yang, X. J. Wu, L. Song, *Proc. Natl. Acad. Sci.* **2023**, *120*, e2217208120.

[2] G. Y. Bai, Z. P. Song, H. Y. Geng, D. Gao, K. Liu, S. W. Wu, W. Rao, L. Q. Guo, J. J. Wang, *Adv. Mater.* **2017**, *29*, 1606843.

[3] S. Zhang, C. Zhang, S. Wu, X. Zhou, Z. He, J. Wang, *J. Phys. Chem. Lett.* **2021**, *12*, 8726-8731.

[4] M. J. Abraham, T. Murtola, R. Schulz, S. Páll, J. C. Smith, B. Hess, E. Lindahl, *SoftwareX* **2015**, *1-2*, 19-25.

[5] M. Matsumoto, T. Yagasaki, H. Tanaka, *J. Comput. Chem.* **2018**, *39*, 61-64.

[6] U. Essmann, L. Perera, M. L. Berkowitz, T. Darden, H. Lee, L. G. Pedersen, *J. Chem. Phys.* **1995**, *103*, 8577-8593.

[7] K. N. Kirschner, A. B. Yongye, S. M. Tschampel, J. Gonzalez-Outeirino, C. R. Daniels, B. L. Foley, R. J. Woods, *J. Comput. Chem.* **2008**, *29*, 622-655.

[8] J. L. F. Abascal, E. Sanz, R. G. Fernández, C. Vega, *J. Chem. Phys.* **2005**, *122*, 234511.

[9] W. Humphrey, A. Dalke, K. Schulten, *J. Mol. Graph.* **1996**, *14*, 33-38.

[10] A. H. Nguyen, V. Molinero, *J. Phys. Chem. B* **2015**, *119*, 9369-9376.

[11] A. Stukowski, *Modell. Simul. Mater. Sci.* **2010**, *18*, 015012.

[12] F. Jiang, X. Zhang, W. Hwang, Y. Nishiyama, R. M. Briber, H. Wang, *Appl. Surf. Sci.* **2021**, *537*, 147783.

[13] Q. Zhang, Y. L. Ma, Y. Lu, L. Li, F. Wan, K. Zhang, J. Chen, *Nat. Commun.* **2020**, *11*, 4463.
